# Supplementary material for: A systematic review of antibody mediated immunity to coronaviruses: kinetics, correlates of protection, and association with severity
Source: Nat Commun. 2020 Sep 17;11:4704. doi: 10.1038/s41467-020-18450-4 (PMC7499300; doi:10.1038/s41467-020-18450-4)
Supplement: Supplementary file 1 — Supplementary Information [file 41467_2020_18450_MOESM1_ESM.pdf]

Supplementary Information

Supplementary Tables

Supplementary Table 1. Summary of Serological Assays.

Assay	Antibody Class	Description	Antigen	Limitations/Comments
Enzyme Linked Immunosorbent Assay (ELISA)	Detects IgG, IgM, IgA	Measures intensity of enzyme-mediated signal of antibody bound to viral antigen.	Viral sub-component or whole virus	Seroconversion not until week 2. Useful for confirming cases that are PCR negative. Cross reactivity with respiratory coronaviruses.
Indirect Immunofluorescence Assay (IFA)	Detects IgG, IgM, IgA	Measures fluorescent activity of antibody bound to viral antigen.	Viral sub-component or whole virus	Compares well with WB and Immunodot, but more expensive. Prone to subjective interpretation, requires BSL3.
Western Blot (WB)	Detect IgG, IgM, IgA	Measures presence of antibody-virus antigen complexes through size exclusion.	Viral sub-component or whole virus	Useful for confirming cases. Synthetic peptides offer improved specificity. Also known as immunoblotting.
Complement fixation (CF)	Aggregate activity	Measures inhibition of a fixed amount of complement to lyse cells by uptake of complement by antibody-virus complexes.	Whole virus	Human sera express an inhibitor mimicking some plaquing factors, leading to false positives. Antibody detected may be time dependent.
Hemagglutination Inhibition (HI)	Aggregate activity	Measures interference of virus-red blood cell binding by antibody binding of virus.	Whole virus	Potential for false positives. Relatively quick, simple, inexpensive.
Neutralization	Aggregate activity	Measures ability of sera to inhibit viral entry and replication.	Whole virus	Gold standard and used often for confirmatory testing. Labor-intensive, expensive, requires BSL3. Can be done in BSL2 when using recombinant pseudotype virus-like particles. Agreement with wild type virus needs to be verified.

Supplementary Table 2. Summary of studies on the kinetics of antibody immunity after infection, and for the association of antibody responses with disease severity.

Author, Year Published	Year of study	Country/ Region	Study type	Participants	Virus	Key findings
Riski & Hovi, 1980[1]	1977 - 1980	Finland	Prospective	Patients with suspected viral infections (n=14,000)	HCoV-OC43	● Suggestive association between high titer to HCoV-OC43 and disease other than common cold, including pneumonia. ● Patients with high or decreasing titer to HCoV-OC43 antibodies could also develop other diseases, including pneumonia.
Kraaijeveld et al., 1980[2]	Appx 1980	United Kingdom	Challenge experiment	Adults (n=15)	HCoV-229E	● Significant antibody rises correlated well with symptoms, clinical score, and virus shedding.
Callow et al., 1990[3]	1990	United Kingdom	Challenge experiment	Adults (n=15)	HCoV-229E	● IgG and IgA antibody levels increased after day 8 in 10 infected individuals. ● IgG and IgA peaked, on average, on day 14. ● IgG and IgA waned but were detectable after 1 year.
Azhar et al., 2014[4]	2013	Saudi Arabia	Case report	Confirmed case (n=1)	MERS-CoV	● First serum sample collected on day 1 was negative for MERS-CoV. ● Serum collected on day 14 detected MERS-CoV antibodies.
Okba et al., 2019[5]	2013	The Netherlands, Qatar, South Korea	Laboratory	Serum from confirmed cases (n=11)	MERS-CoV	● IgG antibodies were detectable and maintained in all severe (n=5) and most non-severe (n=6) cases, after one year, though some lacked detectable neutralizing antibodies. ● Antibody responses tended to be higher among severe cases.

Alshukairi et al., 2016[6]	2014	Saudi Arabia	Retrospective	Survived HCWs (n=9)	MERS-CoV	● Antibodies detected at month 18 in 2 of 9 patients with severe symptoms. ● More variable antibody longevity among patients with milder symptoms.
Spanakis et al., 2014[7]	2014	Greece	Case report	Confirmed case (n=1)	MERS-CoV	● IgG titers peaked 3 weeks after onset of illness, and declined during weeks 4-5. ● IgM titers remained consistently elevated during weeks 2-5.
Park et al., 2015[8]	2015	South Korea	Cross-sectional	Confirmed cases (n=17)	MERS-CoV	● Robust antibody responses by week 3 of illness for most patients. ● Delayed antibody responses with the neutralization test were associated with more severe disease.
Wang et al., 2016[9]	2015	China	Retrospective	Confirmed case (n=1; 52 close contacts)	MERS-CoV	● IgM and IgG levels plateaued at day 15, and neutralizing antibody titer peaked during this time.
Ko et al., 2017[10]	2015	South Korea	Prospective	Confirmed cases (n=42)	MERS-CoV	● No seroconversion among asymptomatic patients (n=3). ● Seroconversion rate grew with increasing disease severity. ● Symptomatic patients without pneumonia (n=10) had a robust increase in antibody titer by week 3. ● Delayed rise in antibodies among patients with pneumonia that progressed to respiratory failure. ● 75% of deceased patients did not seroconvert by week 3, compared to 0% of survivors.

Choe et al., 2017[11]	2015	South Korea	Prospective	Confirmed cases (n=11)	MERS-CoV	● Severe cases tended to have higher antibody responses compared to mild cases.<br>● 5 of 5 patients with severe disease and 2 of 6 patients with mild disease, had detectable antibodies at year 1.<br>● MERS antibodies decreased throughout the 6 months following disease onset.<br>● Antibody titers in 4 of 6 mild cases were undetectable, even if most had pneumonia.
Al-Abdely et al., 2019[12]	2015 - 2016	Saudi Arabia	Prospective	Confirmed cases (n=33)	MERS-CoV	● Patients who died (n=6) exhibited robust neutralizing antibody responses by weeks 2-3, but these were Insufficient for recovery.
Van Kerkhove et al., 2019[13]	2015 - 2016	Saudi Arabia	Cross-sectional	Serum from confirmed cases (n=19)	MERS-CoV	● For all 9 of 19 cases for which a second sample was collected at month 5, IgG antibody levels had waned but were detectable.
Hsueh et al., 2003[14]	2003	Taiwan	Prospective	Probable cases (n=7)	SARS-CoV	● IgG antibodies detected as early as by day 9 in all 6 patients that had detectable antibodies.<br>● Elevated antibody levels lasted from month 1 up to >2 months.<br>● Antibody level plateaued in all patients during days 4-10.<br>● An upsurge of antibody response was associated with the aggravation of respiratory failure.
Wu et al., 2004[15]	2003	Taiwan	Laboratory	Probable cases (n=138)	SARS-CoV	● Antibodies during days 1-7 were detected in 10% (14 of 138) of probable patients.<br>● Proportion of patients that tested positive was 50% at week 3, and peaked at over 70% at week 10.

Chen et al., 2004[16]	2003	China	Prospective	Probable cases (n=36)	SARS-CoV	● Appearance of IgM and IgG ranged from 3-42 and 5-47 days, respectively.<br>● 5.6% of probable infections were still positive for IgG, but negative for IgM up until day 60.
Tsao et al., 2005[17]	2003	Taiwan	Laboratory	Probable cases (n=26)	SARS-CoV	● Antibody titers in five patients were high after 100 days of disease onset.
Chan et al., 2005[18]	2003	Hong Kong	Prospective	Confirmed cases (n=20)	SARS-CoV, HCoV-OC43, 229E	● IgM still detectable in 8 of 11 patients at month 7.<br>● IgGAM and IgG remained stable over the same period.<br>● No significant difference in the kinetics of antibody responses between patients that survived or died.
He et al., 2008[19]	2003	China	Laboratory	Confirmed cases (n=22)	SARS-CoV	● Most sera became positive by day 7.<br>● Inconclusive for differences in antibody responses between recovered and died cases.
Liao et al., 2007[20]	2003 - 2004	China	Retrospective	Confirmed cases (n=18; including 4 reemerging cases)	SARS-CoV	● Neutralizing antibody titers for 14 cases remained high between days 17-181.<br>● Neutralizing antibody titers for all 4 reemerging SARS cases peaked within 11-13 days then rapidly dropped.
Cao et al., 2007[21]	2003 - 2006	China	Prospective	Confirmed cases (n=56)	SARS-CoV	● Titers peaked at month 4.<br>● 100% of participants were seropositive until month 16.<br>● IgG and neutralizing antibodies were undetectable in 19.4% and 11.1% of serum samples, respectively, at month 30, and in 25.8% and 16.1%, respectively, at month 36.

						● Patients with subsequent aseptic femoral neck necrosis had significantly lower neutralizing antibody levels than those without the sequela.
Hsueh et al., 2004[22]	2004	Taiwan	Laboratory	Confirmed cases (n=30)	SARS-CoV	● IgG, IgM, and IgA were detectable for at least 19 days.<br>● On average it took 15 days for all three antibodies to peak.<br>● Levels of IgA and IgM waned during weeks 3-4, remaining low on month 3.<br>● IgG remained positive for > 28 days.
Yang et al., 2009[23]	2004 – 2006 ?	China	Prospective	Confirmed cases (n=67)	SARS-CoV	● 7.7% of samples were positive for IgM after 1 week.<br>● IgM antibodies peaked at month 1 and had a higher positive rate than IgG during this period, followed by a gradual decrease.<br>● IgG levels peaked after week 25, and slowly declined but remained detectable at week 83.
Tang et al., 2011[24]	2020?	China	Prospective	Confirmed cases (n=23; 22 close contacts)	SARS-CoV	● 2 of 23 patients maintained low levels of IgG at year 6.<br>● 1 patient's IgG antibodies were high after being discharged from the hospital, but decreased substantially by month 72.<br>● Another patient's IgG antibody remained low but stable.
Zhang et al., 2020[25]	2020	China	Laboratory	Confirmed cases (n=16)	SARS-CoV-2	● Increase in antibodies was detected in nearly all patients by day 5.<br>● 81% (13 of 16) of patients were IgM positive by day 5.<br>● 100% (16 of 16) were IgG positive by day 5.

Zhao et al., 2020[26]	2020	China	Prospective	Confirmed cases (n=173)	SARS-CoV-2	● Antibody levels increased rapidly during the first 2 weeks.<br>● Cumulative seroconversion reached 100% for IgG and neutralizing antibodies at around month 1.<br>● Seroconversion of neutralizing antibodies was significantly quicker than that of IgM and IgG (possibly also due to the assay used).
Tan et al., 2020[27]	2020	China	Prospective	Confirmed cases (n=67)	SARS-CoV-2	● Positive rate for IgM peaked at 57.1% by day 28, and declined to 33.3% at day 42.<br>● Positive rate for IgG reached 74.3% by day 28 and increased to 86.7% at day 42.<br>● Results suggest that antibody response may be associated with disease severity.
Mo et al., 2006[28]	2002-2003	China	Retrospective	Confirmed cases (n=98; n=18 followed up for 2 years)	SARS-CoV	● IgM, IgG, and neutralizing antibodies not detectable by day 7.<br>● IgM detected day 15, reached peak at month 1, and was undetectable by day 180.<br>● IgG dramatically increased on day 15, peaked on day 60, high until day 180, with gradual decline until day 720.<br>● Neutralizing antibodies increased on day 15, peaked on day 30, and by day 560 and 720, 17 of 18 had low but detectable antibodies
Zhuang et al., 2003[29]	2003	China	Cross-sectional /Prospective	Confirmed cases	SARS-CoV	● IgG was higher than IgM in acute phase (84.6% vs. 2.6%), convalescent stage (100% vs. 80%) and 1-2 months follow-up (100% vs. 20.8%).

Liu et al., 2003[30]	2003	China	Cross-sectional	Confirmed cases (n = 46) HCWs contacting of confirmed cases (n = 64)	SARS-CoV	● IgG among convalescent patients were higher than negative controls. ● IgG among HCWs contacting of confirmed cases were higher than negative controls.

Supplementary Table 3. Severity ratings used in the studies, and the corresponding standardizations.

Author, Year Published	Severity rating used in study	Standardized rating[1]
Kraaijeveld et al., 1980; Alshukairi et al., 2016[2,6]	Asymptomatic	Asymptomatic
Kraaijeveld et al., 1980; Park et al., 2015; Okba et al., 2019[2,5,8]	Mild	Mild
Kraaijeveld et al., 1980[2]	Moderate (HCoV-229E)	
Abbasi et al., 2018[31]	Group 1: Mild - no pneumonia	
Alshukairi et al., 2016[6]	Upper respiratory tract infection	
Choe et al., 2017; Tan et al., 2020[11,27]	Non-severe	
Zhao et al., 2020[32]	Non-critical	
Al-Abdely et al., 2019[12]	Group 1: Received air throughout hospitalization	Severe
Al-Abdely et al., 2019[12]	Group 2: Required ventilator support and survived	
Al-Abdely et al., 2019[12]	Group 3: Required ventilator support and died	
Abbasi et al., 2018[31]	Group 2: Medium - pneumonia, no respiratory failure	
Abbasi et al., 2018[31]	Group 3: Severe - pneumonia and respiratory failure	
Alshukairi et al., 2016[6]	Severe pneumonia	
Choe et al., 2017; Okba et al., 2019; Tan et al., 2020[5,11,27]	Severe	
Park et al., 2015[8]	Severe, requiring supplemental oxygen therapy	
Park et al., 2015[8]	Severe, requiring mechanical ventilation	
Zhao et al., 2020[26]	Critical	

1 We define “mild” as symptomatic cases not requiring hospitalization, and “severe” as symptomatic cases requiring hospitalization. We necessarily made assumptions on what conditions may have likely required hospitalization, e.g., we assume a participant with pneumonia due to a viral infection (and who has been included in these studies) is more likely to have been hospitalized than not.

Supplementary Table 4. Summary of studies on correlates of antibody immunity and protection against CoV infection.

Author, Year Published	Year of study	Country/ Region	Study type	Participants	Virus	Key findings
Callow, 1985[33]	NA	UNITED KINDOM	Challenge experiment	Adults (n=33)	HCoV-229E	● Individuals who seroconverted to 229E (defined as a 1.5 or higher rise in ELISA IgG serum antibodies) had significantly higher serum IgG and neutralizing antibodies as well as nasal IgA.<br>● Serum and mucosal IgA were associated with the duration of viral shedding post experimental infection, with those shedding for 5 days or more having statistically significantly less mucosal IgA than those shedding less than 5 days (0.6 ng/ml versus 4.7 ng/ml, $p<0.01$).<br>● Serum neutralizing antibody was not statistically significantly associated with the duration of viral shedding.<br>● Protective associations of pre-infection serum neutralizing antibody, serum IgG and nasal IgA with clinical severity scores and nasal secretion weights (a measure of severity of rhinorrhea symptoms)
Callow et al., 1990[3]	NA	United Kingdom	Challenge experiment	Adults (n=15)	HCoV-229E	● Volunteers who were infected had lower pre-existing antibodies.<br>● All 5 uninfected volunteers were infected and showed symptoms during the re-infection challenge one year later.<br>● 6 of 9 infected volunteers were re-infected without developing into colds during the re-infection challenge one year later.

Hamre & Beem, 1972[34]	1961-68	United States	Prospective	Medical students<br>(n=12)	HCoV-229E	● 67% (8 of 12) of students with virus isolation had detectable pre-season neutralizing antibodies.<br>● 25% (3 of 12) of medical students who seroconverted to 229E had detectable pre-season neutralizing antibodies.<br>● Pre-season neutralizing antibody level inversely associated with the frequency of significant increase in neutralizing antibody titer after re-infection.<br>● No association between pre-season neutralizing antibody and reinfection determined by CF seroconversion.
Riski & Hovi, 1980[1]	1977-80	Finland	Prospective	Probable cases<br>(n=28)	HCoV-OC43	● Detectable pre-existing CF antibodies were found in 64% (18 of 28) of people who had common cold/pneumonia and increased CF antibodies.<br>● Patients with high pre-existing antibodies or decreasing titer to OC43 antibodies could also develop other respiratory diseases, including pneumonia.
Dijkman et al., 2008[35]	2004	The Netherlands	Prospective, cross-sectional	Newborns and children<br>(n=13 longitudinally; 139 cross-sectionally)	HCoV-NL63, HCoV-229E	● All (n = 13) newborns had maternal antibodies to NL63 and 229E at birth, which disappeared within 3 months.<br>● 53.8% and 13.4% of the newborns seroconverted to NL63 and 229E, respectively.<br>● All newborns who later had seroconversion had low pre-infection antibodies.<br>● 75% and 65.0% of the children aged 2.5 to 3.5 years were NL63 and 229E seropositive, respectively.

Reed[36]	1984	United Kingdom	Challenge experiment	Adults (n=18)	HCoV-229E, HCoV-OC43	● Re-challenged (n = 6) volunteers who had been experimentally infected 8-12 months previously. On the first challenge, all 6 developed symptoms and detectable virus and 5 of 6 experienced significant rise in titer. In the second season, 0/6 experienced illness, detectable virus or significant rise in titer.<br>● Re-challenged (n=12) volunteers with heterologous virus (not identical to first experimental infection) 8-14 months after first infections. 7/12 developed cold symptoms
Cohen[37]	1991	United Kingdom	Challenge experiment	Adults (n=54)	HCoV-229E	● Challenge study focused on psychological-stress and its impact on response to experimental infection with coronavirus.<br>● Suggested that serological status (having above or below median value) was associated with risk but lacks details broken out for just coronavirus.
Barrow[38]	1990	United Kingdom	Challenge experiment	Adults (n=53)	HCoV-229E	● Found lower proportions of individuals with high neutralizing titer experienced ‘significant colds’ upon viral challenge than individuals with low titer.

Supplementary Table 5. Summary of studies on antigenic diversity and cross-reactivity.

Author, Year Published	Year of study	Country/ Region	Study type	Participants	Viruses/assay	Key findings
Bradburne 1970[39]	1970	United Kingdom	Human challenge study	Volunteers challenged with LP (n=18), 229E (n=20), B814 (n=10), OC43 (n=OC43)	HCoV-229E, LP, B814, OC43, neutralization test	● Volunteers inoculated with LP (n=16/18) or 229E (20/20) experienced a >=4-fold rise in neutralizing antibodies to both viruses.<br>● Individuals inoculated with B814 (n=0/10) or OC43 (n=1/14) did not have a >=4-fold rise to 229E or LP.
Kaye et al. 1972[40]	1965-1972	United States	Paired serum in a longitudinal survey of children (=104)	Paired serum in a longitudinal survey of children (=104)	HCoV-OC43, -229E, hemagglutination assay	● Of 104 paired serum of children in a longitudinal survey which showed 4-fold seroconversions by indirect hemagglutination assay (IHA), 41 were risen to only HCoV-OC43, 62 to only -229E, and only one that was risen to both.
Reed, 1984[36]	1974-1976; 1971-1981	United Kingdom	*In vitro and human challenge study*	Volunteers 18-50 yo (n=18) challenged at approximately one-year with the exact homologous strain	HCoV-229E, LP, and 229E-like strains, -OC43 and related strains	● Recent HCoV-OC43-like strains caused different disease manifestations from -229E-like strains did not induce a rise in antibodies (neutralization or HI) to -229E-like viruses or -OC43.<br>● Endemic circulation of HCoV-229E resulted in higher pre-inoculation neutralizing antibodies of new participants against a lab adapted 229E-like strain and decreased clinical disease.

						● Study participants (n=18) challenged at approximately one-year with the exact homologous strain (6/6) were protected, while 7/12 challenged with a heterologous 229E-like strain developed symptoms and shed virus.
Macnaughton et al. 1981[41]	1981	United Kingdom	Human challenge study	Volunteers (n=50) challenged with HCoV-OC43, RO, GI, HO, PA, AD and saline.	HCoV-229E, PR, TO, and OC43, and MHV3, ELISA.	● Antigenic diversity exists within each serogroup (229E-group vs OC43-group).<br>● No cross-reactivity across serogroup.
Dijkman et al., 2008[35]	1999-2003	The Netherlands	Longitudinal serological study	Children born to HIV 1 positive mothers (n=13)	HCoV-NL63 and -299E, ELISA	● Longevity of anti-coronavirus antibodies in newborns and cross reactivity. Did not find cross-reactivity between HCoV-NL63 N-direct antibodies and -229E N-direct antibodies.
Dijkman et al., 2012[42]	1993-2012	The Netherlands and United Kingdom	Serological Survey	(n=12 males and n=13 females) born to HIV 1 positive mothers (NL63 and 229E in infants followed 20 months (n=25)) hospitalized infants (n=1471)	ELISA to N protein, NL63 and 229E in infants followed 20 months (n=25) and hospitalized infants (n=1471)	● Anti-HCoV-NL63 neutralizing antibodies against the spike protein may partially protect against HCoV-229E but not the vice versa. This is also the case for antibodies to HCoV-OC43 and protection against HCoV-HKU1. This may also account for the higher frequency of HCoV-OC43 and -NL63 among hospitalized infants.

Lehmann et al., 2008[43]	2008	Germany	Serological Assay	Acute and convalescent sera (n=6 HCoV-OC43 infection, n=6 HCoV-229E infection)<br>n=25 healthy donor sera<br>n=49 sera from convalescent SARS sera	HCoV-229E, OC43, HKU1, NL63, SARS-CoV, developed line immunoassay	● Supports cross-reactivity among endemic strains within alpha- and beta-CoVs, but not with SARS-CoV N protein.
Haynes et al., 2007[44]	2007	United States	Comparison of serological assay detection of N and S proteins	Baby hampster kidney and n=61 patients from Vietnam and Taiwan SARS-CoV positive	SARS-CoV, ELISA	● Found false-positive to SARS N/S recombinant ELISA but unknown whether it is due to cross-reactivity with other CoVs or are they just nonspecific reactivity.
Che et al., 2005[45]	2003-2005	China	Serological study	n=11 paired serum samples from patients with SARS-CoV, n=100 random samples were collected from healthy adult donors, n=34 SARS-CoV patients 8-81 days after onset	SARS-CoV, IFA, Western Blot, ELISA	● Only 2/100 healthy donor samples had SARS-CoV nucleocapsid reactivity, as compared to 97% with positivity to HCoV-229E and 99% positivity to HCoV-OC43.<br>● SARS patients (n=34) had strong reactivity to nucleocapsid from HCoV-229E (97%), HCoV-OC43 (100%) and SARS-CoV (100%). Similar trends were observed when instead the samples were tested by IgG responses to CoV-infected cells.<br>● 10/11 SARS patients showed >=4-fold rise to HCoV-OC43 and 5/11 to 229E by IFA in paired acute/convalescent samples, but more limited seroconversion to the nucleocapsid of -229E (2/11) and -OC43 (0/11).

Chan et al., 2013[46]	2013	Hong Kong	Serological study	Animal handlers (n=94), SARS-CoV patients (n=28), healthy blood donors (n=152)	SARS-CoV, IF and neutralization	● 100% of SARS-CoV patients had IF titers >1:10 and 96.4% had neutralizing titers >1:10 against SARS-CoV, while 60.7% had IF titers and 25% had neutralizing antibodies to MERS-CoV.<br>● The proportion of cross-reactivity was lower for animal handlers at high risk of exposure to SARS-like CoVs (SARS-CoV: 13.8% IF >1:10, 4.3% with NT >1:10; MERS-CoV: 2.2% IF >1:10, 0% NT >1:10).<br>● None of the healthy donors had any reactivity to either MERS-CoV or SARS-CoV.<br>● Among animal handlers and SARS patients with positivity to MERS-CoV by IF, 7/19 had NT to MERS-CoV. All had high levels of NT to OC43. SARS-CoV patients with paired acute/convalescent sera (n=4) experienced seroconversion by (IF and NT) to SARS-CoV; some had positivity by IF to MERS-CoV in either acute (n=1) or convalescent (n=2) samples. All showed >2-fold rise to betacoronavirus OC43 by IF, while only 1 seroconverted to alpha coronaviruses 229E and NL63 by IF.
Chan et al., 2005[18]	2003-2005	Hong Kong	Serological cohort study	n=20 SARS-CoV patients in the first month of illness. Patients who survived (n=14), patients who died (n=6)	SARS-CoV, HCoV-229E, -OC43, -NL63, IFA and neutralization	● Infections with HCoV-OC43 and HCoV-229E did not lead to antibodies (acute or convalescent phase) against SARS-CoV by IFA or neutralization.

						● Total Ig (IgG, IgA, and IgM) and IgG to endemic HCoVs (-229E, -OC43) measured by IF showed a 4-fold rise after SARS-CoV infection in 12/20 patients. A subset also had a rise in antibodies to NL63.<br>● Neutralization titers to SARS-CoV remained stable for 7 months.
Liang et al., 2005[47]	2005	China	Mouse challenge study	Generated 14 mAbs against SARS-CoV N protein.	SARS-CoV, IFA	● Generated mAbs against SARS-CoV N do not crossreact with N from 229E and OC43 by IFA<br>● Identified immunogenic epitopes and whether they were linear or structural
Fung & Liu, 2019[48]	2019	China	Review	Vaires	Human coronaviruses, various	● Review study of of human coronavirus pathogenesis
Zhong et al., 2005[49]	2003-2005	China	Serological cohort study	n=40 patients who recovered from SARS-CoV 1 month after discharge (20-65 yo)	SARS-CoV, ELISA, Western Blot	● While most neutralizing antibodies against CoVs target epitopes in the S1 region, the only identified surface immunodominant site in a study identifying immunogenic epitopes from convalescent samples of twenty SARS survivors through biopanning against phage display dodecapeptide library resides in the S2 domain.
Cui et al., 2019[50]	2019	United States	Review	Varies	Human coronaviruses	● Review on coronavirus evolutionary history
Ren et al., 2003[51]	2003	China	Serological study	SARS-CoV sera (n=4)<br>Healthy sera (n=2)	SARS-CoV, ELISA	● Characterized antigenic regions on recombinant S1 and S2 protein by Western Blot and ELISA

Bisht et al., 2004[52]	2004	United States	Mouse challenge study	2 groups of 8 BALBc mice 0-4 weeks received MVA/S or MVA i.m.	Attenuated modified vaccinia virus Ankara (MVA) and SARS-CoV, Western Blot, ELISA, neutralization	● Characterization of the SARS-CoV S protein
Wang et al., 2005[53]	2005	Taiwan	ADE and cell infectivity study	Anti-SARS-CoV sera were collected from SARS-CoV patients	Immunoblotting and RT PCR	● Investigated the timing of IgG reactivity up to and after 3 weeks demonstrated stronger immunogenicity/antigenicity of N and S3 protein compared to S1 and S2.
Meyer et al., 2014[54]	2014	Germany	Serological assay development Review	Varies	HCoV-229E, -NL63, OC43, HKU1, SARS-CoV, MERS-CoV,IFA, ELISA, Western Blot	● Review of serological assays for SARS-CoV, MERS-CoV, and newly emerging CoVs
Patrick et al., 2006[55]	2003-2006	Canada	Serological study	n=95/142 residents n=53/160 staff experienced symptoms of SARS-CoV	SARS-CoV, RT PCR, IFA, ELISA, neutralization, Western Blot, Euroimmun indirect	● A study screening 220 ten amino acid peptides covering the full length of HCoV-OC43 N protein (with eight residues running overlaps) and 207 peptides of SARS-CoV N revealed four sites with shared homology ● The study was conducted to explain false-positives to SARS-CoV in multiple assays. Supported by non-seroconversion in the neutralization assay and RT-PCR positive results for HCoV-OC43, these were likely HCoV-OC43 infections with cross-reactive results driven by these potentially cross-reactive sites.

Du et al., 2013[56]	2013	United States and China	Serological study	SARS-CoV S-RBD protein-vaccinated mice	SARS-CoV, neutralization	● Find whether the cross-reactivity between SARS-CoV and MERS-CoV was due to antibodies targeting the RBD. They found monoclonal antibodies raised to SARS-CoV RBD did not bind the MERS-CoV RBD even at high concentrations (10ug/mL) and all had low or no neutralizing activity against MERS-CoV pseudoviruses.<br>● There is an absence of cross-neutralization of MERS-CoV isolates by antiserum to S glycoprotein HKU 4 or 5 (animal CoVs in the same subgroup). For SARS-CoV, there is also an absence of cross-neutralization by antiserum to HKU 3 and BtCoV 279 S glycoprotein.<br>● Between SARS-CoV and MERS-CoV, all mAbs generated that have high affinity to conformational or linear epitopes in the RBD of SARS-CoV does not neutralize nor bind to the RBD and S1 proteins of MERS-CoV even at high concentrations (10 mg/ml).

Aburizaiza et al., 2014[57]	2012	Saudi Arabia	Serological study	healthy individuals (n=130) slaughterhouse workers (n=226)	MERS-CoV, IFA and neutralization	● None of the healthy individuals had IFA positivity to MERS-CoV, while 8/226 slaughterhouse workers had some cross-reactivity by IFA to MERS-CoV and 2 had reactivity to the MERS-CoV spike protein, as well as some reactivity to 229E, NL63, OC43 and/or HKU1 in immunofluorescence assay (IFA). None had a response to the SARS-CoV spike protein, although, antisera from confirmed MERS patients in Germany high PRNT90 titers (1:320 and 1:640) to MERS-CoV showed slight cross-reactivity with SARS-CoV by IFA.
Al Kahlout et al., 2019[58]	2012-2016	Qatar	Serological Study	4858 plasma samples (n=4719 blood donors 19-88yo), (n=135 close contacts to 4 confirmed cases 14-49 yo), (n=4 confirmed patients 30-70yo)	recombinant S1 protein IgG ELISA kit	● Low positivity among healthy blood donors (10/4719 tested, n individuals ages 19-88 years), contacts of confirmed MERS patients (1/135 tested, 14-49 years; mean age 31 years), while 3/4 confirmed MERS cases were positive (30-70 years; mean age 52), but random testing of the blood donors using an IgM assay revealed some false negative results, suggesting the assay was underestimated prevalence. Blood donors that were positive and negative for MERS antibodies had high prevalence of antibodies to HCoV-229E, -NL63, -OC43, and -HKU1.

Gao et al., 2015[59]	2015	Turkey and United Kingdom	Seroprevalence study	n=695 healthy adults and n=492 healthy children for seroprevalence and to test the relationship between anti-N-IgG and HCoV infection n=361 serum samples from children with LRI were used	HCoV-229E, -NL63, -OC43, -HKU1, SARS-CoV, and MERS-CoV Western Blot, ELISA	● Reactivity of human positive control antisera to each of the various strains (HCoV-NL63, -229E, -OC43, -HKU1, SARS-CoV, MERS-CoV) as well as antiserum to EV68 as negative control were measured against nucleocapsid (N) of these strains across a gradient of concentrations. SARS-CoV showed no cross-reactivity. Comparing the reactivity patterns, HCoV-HKU-1 appeared antigenically close to -OC43, and -229E appeared close to -NL63, but the distance was non-symmetric.
Trivedi et al., 2019[60]	2019	United States	Development and Evaluation of Immunoassay	Positive for HCoV-229E (n=4), HCoV-NL63 (n=9), HCoV-OC43 (n=21), HCoV-HKU1 (n=14), SARS-CoV (n=5), MERS-CoV (n=7)	HCoV-229E, HCoV-NL63, HCoV-OC43, HCoV-HKU1, SARS-CoV, MERS-CoV, RT PCR, ELISA, multiplex immunoassay	● Measured the mean fluorescent intensity (MFI) of reactions between positive control sera and recombinant N of those strains. Group 1 HCoVs (alpha-HCoV-229E and -NL63) cross-reacted with other group 1 CoVs but did not cross-react with strains from group 2 (beta-HCoV-OC43 and -HKU1). Antiserum against SARS-CoV (from group 2b) reacted with HCoV-229E and -NL63. Antiserum against HCoV-OC43 (from group 2a) reacted with -HKU1 (also in 2a). Antiserum against MERS-CoV (group 2c) reacted with HCoV-HKU1.

Agnihothram et al., 2014[61]	2012-2014	United Kingdom	Serological Study	MERS-CoV was isolated from a 49yo patient	MERS-CoV, ELISA, neutralization	● Antiserum against MERS-CoV (group 2c) reacted with HCoV-HKU1 were consistent with cross-reactivity of epitopes in the N protein between subgroups but not between those observed in polyclonal sera generated through mice immunization.<br><br>● It is unclear how much of the in vitro fitness and plaque morphology differences can be attributed to the antigenic differences observed between strains.
Vlasova et al., 2007[62]	2007	United States	Serological study and protein reactivity	SARS patients (n=6, convalescent samples collected 18-50 days post-symptom onset)<br>healthy human samples (n=24)	SARS-CoV, s, HCoV-NL63, ELISA, Western Blot	● Examined cross-reactivity (binding by ELISA and Western Blot) among CoV N proteins between SARS-CoV N and animal CoV N proteins (porcine CoVs gastroenteritis CoV [TGEV] and porcine respiratory CoV [PRCV], feline infectious peritonitis virus [FIPV], and canine CoVs).<br><br>● Found cross-binding by serum from SARS patients against SARS-CoV and porcine CoVs N protein sites while healthy human samples showed no binding to SARS-CoV porcine CoV N proteins. Both groups had high reactivity to HCoV-NL63.

Yu et al., 2005[63]	2003-2005	Japan and Vietnam	Serological study	n=149 healthcare workers, 37 probable SARS-CoV cases<br>n=175 healthy volunteers	SARS-CoV, ELISA, Western Blot, neutralization	● Showed nonspecific reaction to N protein by ELISA was reduced through the use of N constructs with 121 amino acid deletions at the N-terminus compared to four residue deletions.<br>● When tested against sera from healthy volunteers and SARS-CoV patients in Vietnam, the resulting titers were higher than that of SARS-CoV-infected cell lysate-based ELISA. Of those include four inapparent SARS-CoV infections confirmed by virus neutralization.
Mu et al., 2008[64]	2003-2008	China	Serological challenge study	SARS patients (n=457/460+, 35-114 days post-symptom onset)<br>healthy donors (n=650/650-)	Immunoblot analysis, IFA, ELISA, neutralization test,	● Explored recombinant S and N protein as diagnostic tools for identifying SARS-CoV patients and found an ELISA testing positivity to a truncated S-N protein (N321-422 and S264-680) proteins from SARS-CoV could discriminate between SARS-CoV patients and healthy donors, nearly as well as SARS-CoV lysate and better than S or N alone. SARS-CoV patients showed more positivity to full N proteins of HCoV-229E and -OC43 than truncated versions of N.

Liang et al., 2013[65]	2013	Taiwan	Serological study	n=26 human serum samples from young healthy adults 18-26 yo, n=17 serum samples people 50-80 yo who reported to the hospital with respiratory tract infection, n=15 cord blood samples	HCoV-OC43, other human coronaviruses (HCoV-229E, SARS-CoV) Western blot	● No cross-reactivity with SARS-CoV nor HCoV-229E was observed. Immunoblotting assessment on three structural regions of the NP showed strongest reactivity at the central-linker region (aa174-300) followed by the C-terminal domain (aa301-448), and low at the N-terminal domain (aa1-173). Western blot assay against the recombinant protein in sera from adults (92.3% positive), patients with respiratory infection symptoms (82.3%), and cord blood samples (93.3%), showed no reaction to SARS-CoV NP but 81% reacted to HCoV-229E NP.<br><br>● The reaction (quantified in 38 of the 52 HCoV-OC43 positive sera) to the three structural regions showed three distinct patterns. All patterns reacted highly to the central-linker region, another pattern was also reactive to the C-terminal region, and the last pattern strongly reacted to all.
Carattoli et al., 2005[66]	2005	Italy and Germany	Serological assay development (protein-based)for SARS-CoV diagnosis	n=6 SARS-CoV Controls= (n=20 healthy donors) and (n=73 patients with non-SARS-CoV infections)	SARS-CoV, ELISA, immunocytochemical assay	● Membrane protein could be used to distinguish serum from SARS-CoV patients who had strong responses and low inter-subject variability in responses to the M2 antigen. Healthy controls did not show a response.

He et al., 2005[67]	2003-2005	United States and China	Serological study recognizing epitope	n=40 convalescent SARS-CoV patients 30-60 days after onset, n=30 healthy blood donors	SARS-CoV, ELISA	● Showed the membrane protein can induce antibodies in experimental animal inoculation.
Rockx et al. 2008[68]	2008	United States	Mouse challenge study	Passive immunization was conducted on 10-12 week old BALBc mice<br>Human monoclonal antibody generation were described in:Traggiai et al. (2004)	SARS-CoV, Neutralization, ELISA,	● Found escapes from mAbs targeting the spike receptor-binding domain (RBD) of SARS-CoV.
Zhu et al. 2007[69]	2007	China	Immunogenicity study	n=6 bats showing positive or negative cross-reactivity with SARS-CoV	SARS-CoV, HIV-pseudotyped S proteins (SARS-CoV), ELISA, neutralization	● While investigating broadly neutralizing antibodies, escapes from mAbs targeting the spike receptor-binding domain (RBD) of SARS-CoV were observed from zoonotic (palm civets and bats) to early to late human strains.
Liu et al., 2007[70]	2002-2007	China	Phylogenetic and cross-neutralization study	Convalescent sera 3-12 months post-recovery (n=20) in SARS-CoV pseudovirus study<br>Full-length civet S gene Immunization of 6-8 week BALBc mice<br>Other convalescent sera collected 2002-2003 at 3-12, 24 month post-recovery	SARS-CoV, pseudovirus for contransfecting viral entry assay, neutralization assay and civet viruses	● Sera from BALB/c mice immunized with full length S protein in civet strains were ineffective against human SARS-CoV and vice versa.

He et al., 2006[71]	2002-2006	United States	RBD study	SARS-CoV isolates from both 2002-2003 and 2003-2004 outbreaks and palm civet isolate SZ3<br>Immunized mice and rabbits	SARS-CoV, ELISA, neutralization	● Single mutations were shown to disrupt neutralizability even though there was significant cross-reactions between mAbs against conformational epitopes of RBD with multiple mutational differences.
Elshabrawy et al., 2012[72]	2012	United States	Immunotherapy study	Produced and characterized human monoclonal antibodies that neutralized SARS-CoV by binding the S2 protein	SARS-CoV, and pseudovirus generated with methods from (Coughlin et al. 2007)<br>ELISA	● Monoclonal antibodies targeting regions critical for fusion and entry on the S2 protein appeared broadly neutralizing against SARS-CoV strains.
Tian et al., 2020[73]	2019-2020	China	RBD study	Antibody CR3022 isolated from convalescent SARS-CoV patient	SARS-CoV-2, SARS-CoV, MERS-CoV<br>Biolayerinterferomtry binding assay, ELISA	● Differential binding of monoclonal antibodies from SARS patients to the receptor binding domain (RBD) of SARS-CoV and SARS-CoV-2.<br>Differences between SARS-CoV and SARS-CoV-2 are largely located at C-terminus residues of the RBD, with structural differences that affect sensitivity to neutralizing antibodies but does not diminish the ability to bind to the ACE2 receptor (suggested by sequence analysis).<br>One antibody potently bound to SARS-COV-2 RBD protein but did not compete with the RBD for binding to the ACE2 receptor or with other RBD-directed antibodies for binding to the RBD

Tai et al., 2017[74]	2012-2017	China	RBD Study	n=20 human and camel isolates from GenBank database	MERS-CoV and MERS-pseudovirus, Western blot, coimmunoprecipitation assay, ELISA,	● Five recombinant receptor binding domains (rRBD) proteins were constructed with mutations detected from MERS-CoV strains detected in humans (2012-2015) and camels.<br>● These rRBDs maintain functionality and do induce potent neutralizing antibodies. When residues in their receptor binding motifs were mutated to evade neutralization, cross-reactivity persisted but binding affinity to DPP-4 was lost suggesting limited antigenic escape for MERS-CoV. 2/29 amino acid differences between MERS Eng1 isolated from a patient transferred to London and MERS SA1 from Saudi Arabia were on the S glycoprotein.
Yu et al., 2007[75]	2007 Vaccine produced 2002-2003	China	Pooled vaccine study	Pooled positive sera from people with SARS-CoV Antisera from group of inbred mice immunized with childhood vaccines	SARS-CoV, ELISA	● Evaluated whether antibodies (binding and neutralizing) and T cell responses induced by childhood vaccination (AMPV, BCG, DPT, HBV, HIB, JEV, MMRV [and MV, RV], OPV, PI, SV, VV [varicella vaccine]) cross-reacted with SARS-CoV. They found no cross reactivity in any assay. They did find serum from children with prior SARS-CoV infections cross-reacted to vaccine antigens, likely because the children received these vaccines.

Resta et al., 1985[76]	1982-1985	United States	Isolation study	Stool samples from infants with necrotizing colitis (n=12) and<br>Control samples (n=14)	Human enteric coronavirus, HCoV-OC43 and -229E, mouse hepatitis virus, ELISA	● Infants with necrotizing enterocolitis were found to have coronavirus particles by electron microscopy. Serum from infants did not cross react with common human CoVs (OC43, 229E), mouse hepatitis virus (MHV) or strains identified as Breda 1 and 2.
Gerna et al., 1984[77]	1984	Italy	Antigenic relatedness study	acute gastroenteritis (n=34/208)<br>age-matched controls (n=3/182)	Human enteric coronavirus and HCoV-OC43	● Identified greater prevalence human enteric coronaviruses in infants with acute gastroenteritis compared to age-matched controls and that virus and antiserum showed antigenic cross-reactivity (immune electron microscopy) to HECV-24, HECV-35, and HCoV-OC43.
Han et al., 2006[78]	2006	United states	Challenge study	Child with acute diarrhea, gnotobiotic calves (n=4)	Human enteric coronavirus and bovine enteric coronavirus, ELISA	● Identified an HCoV from a child with acute diarrhea as a variant of a bovine CoV.
Gerdes et al. 1981[79]	1980-1981	United States	Serological cross-neutralization study	n=2 multiple sclerosis patients	HCoV-229E and -OC43, plaque assay and plaque neutralization	● Two HCoV strains extracted from brain tissue of multiple sclerosis patients were cross-neutralized by antiserum against HCoV-OC43 but not -229E.

Kaye et al., 1977[80]	1960-1977	United States	Serological assay study	n=345 sera from adults with and without respiratory illness, n=213 sera were from adult men in a chronic bronchitis study, n=88 acute and convalescent sera with URI, n=44 sera from influenza vaccine study<br>n=104 acute and convalescent serum pairs from children in longitudinal study of respiratory illness	HCoV-229E and -OC43, indirect hemagglutination, and CF	● Sera positive for HCoV-OC43 were also positive for hemagglutinating encephalomyelitis virus (HEV) while HCoV-229E and HCoV-B814 positive sera were not. Individuals with and without possible contact with swine had significant differences in their HCoV-OC43 and HEV responses while sera collected from swine with Abs to HEV were not positive for HCoV-OC43.<br>● Suggest cross-reaction between the two viruses but did not preclude the possibility of uncharacterized CoV.
Schmidt & Kenny, 1981[81]	1981	United States	Immunogenicity and Antigenicity study	Paired sera (acute collected close to onset and convalescent 25-58 days later) from pneumonia patients	HCoV-229E and -OC43, CF, HI, neutralization	● Three different antigens for coronaviruses.<br>Samples from pneumonia patients had high reactivity to the 'slow' migrating antigen (Two-dimensional immunoelectrophoresis) of HCoV-OC43 than -229E (probably spike) suggesting it was highly immunogenic.

McIntosh et al., 1970[82]	1965-1970	United States	Seroepidemiological study	Acute sera and nasopharyngeal washing from NIH employees with respiratory tract disease on or before fourth day of illness and coronavirus convalescent sera three weeks later (n=466)<br>Throat and nasal swabs from pediatric patients on admission and three weeks later (n=565)<br>Human embryonic tracheal organ cultures were used for diploid cell culture	HCoV-229E, -OC43, -OC38, and MHV, CF	● The highest percentage of dual responses (n=91) involved children with -OC38 and -OC43 in coronavirus antigen tests. Some overlap between MHV and -OC38 and/or -OC43. No or few dual response with -229E and MHV or -229E and -OC38 and/or -OC43
Monto & Lim, 1974[83]	1966-1974	United States	Prospective study	Families where parents were under 46 yo	HCoV-OC43CF and HAI	● Showed low agreement between CF and HI for HCoV-OC43 in sync with a large outbreak of HCoV-229E. Agreement was higher in 1966 and late-1968 to 1969 and showed increased agreement with neutralization test results in selected subsets (66.7%) compared to 28.6% in the other time periods.
Tuan et al., 2007[84]	2003-2007	Vietnam	Retrospective cohort study	n=63 SARS-CoV cases (n=53 primary cases, n=37 healthcare workers)<br>n=252 close contact of primary 45 cases	SARS-CoV, ELISA and RT PCR	● Cross-reactivity between HCoV-OC43 and SARS-CoV was found in one individual while cross-reactivity between HCoV-229E and -OC43 were not found.

Richardson et al., 2004[85]	2004	Canada	Diagnostic review	varies	SARS-CoV, review of assays and diagnostics	● Immunological cross-reactivity has not been detected in SARS-CoV and coronaviruses in antigenic groups 1 and 2. However, after antibody testing for an outbreak of HCoV-OC43, some positive results for SARS-CoV suggest there may be cross-reactivity. This may impact assay development.
Severance et al., 2008[86]	2008	United States	Seroprevalensce and immunoassay study	n=196 between 18-65 yo served as control Seropositivity assay cutoffs were determined from n=10 seronegative children from a vaccine study	HCoV-229E, -HKU1, -NL63, -OC43, and feline coronavirus ELISA	● Correlations between antibody levels with the highest association were within the same group.<br>● Cross-reactivity in this study may be due to using the whole nucleocapsid sequence. However, because there was an absence of reactivity to the feline coronavirus this cannot be fully explained by groups and may be due to exposure to the particular virus.
Kossyvakis et al., 2015[87]	2014-2015	Greece	Laboratory investigation	An imported case of MERS-CoV to Greece (69 yo male)	MERS-CoV, RT PCR	● Implications for receptor binding efficiency were found in unique amino acid substitution in the spike receptor binding domain.

Supplementary Table 6. Summary of studies on immunopathogenesis.

Author, Year Published	Year of study	Country/ Region	Study type	Participants	Viruses/assay	Key findings
Peiris et al., 2003[88]	2003	Hong Kong	Prospective study	Residents of a housing estate. Patients meeting WHO SARS definition (n=75)	SARS-CoV, RT PCR	● The timing of IgG (starts approximately day 10) is linked to decreasing viral load but severe clinical worsening. Report enhancement of Abs within episode. This points to the host's response and not viral replication and ADE.
Ho et al. 2005[89]	2003	Taiwan	Retrospective study of patient data	All patients with probable SARS-CoV (n=665) with (n=347) positive cases	SARS-CoV, RT PCR and ELISA	● Longer hospital stays and death were associated with early seroconversions of neutralizing Abs to SARS-CoV spike protein, week 5 vs week 8 post fever onset. Worsening pulmonary function was associated with decreasing viral load. This plus clinical studies on cytokines, suggests activation of the Th1 cell-mediated immunity and a hyper-innate inflammatory response lead to severe infection. Suggests ADE and priming effect from existing antibodies against endemic strains of coronavirus.
Hsueh et al., 2003[14]	2003	Taiwan	Serological study	Hospitalized patients (n=7, n=6 male) who met the CDC and WHO case definition for SARS-CoV	SARS-CoV, IFA, RT PCR	● There was an upsurge of IgG antibodies, which correlated with ARDS progression. This may be due to host response rather than viral load and suggests ADE.

Cameron et al., 2008[90]	2008	Varies	Review	Varies. One study cited included well-defined SARS-CoV patients from Toronto (n=50)	SARS-CoV	● Expression of a group of proinflammatory cytokines and chemokines is associated with acute and possibly progressing SARS.<br>IFN and ISG are critical in SARS clinical evolution.<br>Non-severe, severe, and fatal patients expressed differences IFN and ISG compared to healthy controls.<br>The study supported prolonged levels of proinflammatory chemokines due to absence of effective adaptive response for virus clearance but the mechanism which led to the malfunctional switch between innate and adaptive is unknown.
Talbot et al., 2009[91]	1977-2001	United States	Longitudinal cohort study	children <5 years old LRI (1977-2001) (n=1830, 3958 child-year) (n=948 LRI), (n=553 nasal wash specimens)<br>URI (1982-2001)(n=1481 children and n=6724 episodes) (n=2082 URI specimens)	HCoV-229E, -NL36, and -OC43, RT PCR	● RSV-associated LRI occurs in children <6 months while LRI with HCoV was in children 6-23 months. This may be due to the presence of maternal antibodies in the LRT or immunopathogenesis.<br>URI infection burden was close to uniform with regard to age.

Jaume et al., 2011[92]	2011	Hong Kong	*In vitro study*	Various cell lines and 6-8 week old BALB/c mice (n= 4 to 5 per group)	SARS-CoV, IF, ELISA, neutralization, and RT PCR	● Anti-Spike immune serum inhibited viral entry in permissive cell lines but potentiated infection of immune cells by SARS-CoV particles.<br>Antibody-mediated infection was dependent on Fc-gamma-RII but did not use the pathway used by ACE2.<br>None of the non-neutralizing responses elicited towards different immunogens involved IgG2a.<br>ADE for SARS-CoV uses a novel entry method into immune cells.
Yip et al. 2014[93]	2014	Hong Kong	*In vitro study*	Healthy samples were collected from Hong Kong Red Cross Blood Transfusion Service and BALBc mice were immunized	SARS-CoV, IF, ELISA and RT PCR	● Human macrophages can be infected by SARS-CoV as a result of IgG-mediated ADE. This indicates that this infection route requires signaling pathways activated downstream of binding to FcγRII receptors.<br>In monocytes, macrophages, and monocyte-derived dendritic cells, virus replicates up to six hours but does not exit the cell.
Fu et al., 2020[94]	2020	Varies	Review	Varies	SARS-CoV and 2 and HNL36	● In a SARS-CoV macaque model it was found that S-IgG present in lungs can cause severe lung injury. In vaccinated macaques acute lung injury was more pronounced than those unvaccinated.<br>This suggests that ADE may be why patients who produce neutralizing antibodies early experience persistent inflammation, ARD, and succumb to SARS-CoV.

Wang et al., 2014[95]	2014	Taiwan	*In vitro study*	Anti-SARS-CoV sera was collected from SARS-CoV infected patients in Taiwan BALBc mice were immunized	SARS-CoV, IF and RT PCR	● Upon infection, TNF-alpha, IL-4 and IL-6 expressions increased while only trace amounts of IL-3 and IL-1beta appeared.<br>Mild to moderate enhancement by various anti-S1a and anti-S1b murine mAbs generated except one particular anti-S1b clone that neutralized it. No effect was seen for anti-N mAbs.<br>ADE is mediated by diluted antibodies against envelope spike proteins not nucleocapsid proteins.<br>HL-CZ cells express ACE2 receptors and display a cytopathic effect caused by SARS-CoV.
Yip et al., 2016[96]	2009-2011	Hong Kong	*In vitro study*	6-8 week BALBc mice were immunized (n=4-5 per group)	SARS-CoV, IF and RT PCR	● Antibody-dependent enhancement (ADE) allows SARS-CoV to infect primary human macrophages, but it does not sustain productive viral replication in the infected cells.<br>● ADE of SARS-CoV infection does not change pro-inflammatory gene expression profile of primary human macrophages.<br>TNF-alpha, IL-4 and IL-6 expressions heightened while IL-3 and IL-1beta only appeared in trace amounts. This could be due to the cell line differences or the set of mediators assessed.
Perlman & Dandekar, 2005[97]	2005	Varies	Review	Varies	SARS-CoV	● Prolonged tissue destruction can heighten presentation of host proteins to T- or B-cells and result in adaptive response against self, i.e. epitope spreading.

Lin et al. 2005[98]	2003	Taiwan	Serological study	Sera was collected from SARS-CoV positive patients (n=80 < 20 days after fever onset) and n=41 >=20 day after fever) n=10 controls	SARS-CoV, Preabsorption and binding assays, ELISA	● Autoantibodies reacted with A549 cells Evidence of pathogenesis when anti-S2 Abs in SARS-CoV cause cytotoxic injury as well as enhanced immune cell adhesion to epithelial cells.
Sun et al., 2010[99]	2003-2009	China	Serological study	The study quantified titers of IgA, IgM, IgG of these Abs in 62 post-SARS osteonecrosis patients and 52 healthy individuals.	ELISA to detect anticardiolipin antibodies	● Though post-SARS patients more associated with at least one of these Abs (33.9% vs 7.7%), no post-SARS patients without osteonecrosis was studied for comparison. The mechanism of pure discussion. No evidence was provided.
Fang et al. 2010[100]	2003	Taiwan	Proteomic study	Sera was collected from SARS-CoV positive patients (n=5 late stage >=20 days after fever onset)	SARS-CoV, ELISA, IF, WB	● Evidence of SARS-CoV pathogenesis was found in upregulated expression of annexin A2 by SARS-associated cytokines and the cross-reactivity of anti-SARS-CoV S2 antibodies to annexin A2.
Cheng et al., 2005[101]	2005	Hong Kong	*In vitro study*	The SARS-CoV genomic library was used and mice were infected	SARS-CoV, Phage ELISA	● Evidence of anti-N antibodies that cross react with IL-11 including lung and bone marrow.<br>High anti-N antibodies induced relatively early during infection may be involved in the thrombocytopenia and lymphopenia observed early in SARS-CoV infection.

Yasmon et al., 2012[102]	2012	Germany	Serological study	n=20 health sera were used as control<br>n=129 (n=61 HIV-1 negative and n=68 HIV-1 positive) were collected from IDU in Jakarat, Indonesia<br><21 yo	SARS-CoV and HIV,<br>IgG ELISA	● Show anti-N antibodies that cross react with IL-11.
Ksiazek et al., 2003[103]	2003	United States	Serological Study	Clinical specimens=serum from SARS-CoV patients in Singapore, Bangkok, and Hong Kong (n=19).<br>Healthy blood from the US CDC<br>And patients with known OC43 and 229E	Group 1 coronaviruses, SARS-CoV<br>RT PCR, IFA, ELISA, suckling ICR mice were used to isolate virus	● Did not show cross-reactivity with the same immune human serum sample and feline infectious peritonitis virus 1 antigen.<br>Paired human serum samples with diagnostic increases (by a factor >=4) in antibody (with very high titers to the homologous viral antigen in the convalescent-phase serum) to the two known human coronaviruses, HCoV-OC43 (13 pairs) and -229E (14 pairs), showed no re-activity in either acute- or convalescent-phase serum with the newly isolated coronavirus.

Gorse et al., 2020[104]	2009-2013		Serological study	Group 1: 99 of age>=60 with underlying chronic lung and heart disease; Group 2: 101 healthy adults of age 21-40 years old	HCoV-229E, -NL63, -OC43	● Baseline binding antibody titers to all strains (by ELISA) were higher in the older adults. Post infection, neutralizing Abs were more efficiently triggered in the older group to HCoV suggesting the role of cross-reacting Abs from past exposures.<br>Antibody-dependent enhancement of SARS-CoV and feline infectious peritonitis virus infectivity has been reported and can be mediated by antibodies to S protein epitopes. studies of the S protein sequence and neutralization antigenicity suggest that serum antibodies may not cross-react as well with the laboratory strains of HCoV that were used in our neutralization assays, affecting the sensitivity of the neutralization assay used in the study.
Bermingham et al., 2004[105]	2004	United Kingdom	Review	Various	Review of molecular detection, targets, diagnostics, and assays	● Demonstrates limited cross reactivity with antibodies to human group 1 or group 2 coronaviruses and group 1 animal coronaviruses
Yang et al., 2005[106]	2004	United States and Switzerland	Neutralization test	Purified immune IgGcame from vaccine candidates<br>5 female BALBc mice per group (6-8 weeks old)	SARS-CoV and hACE-2, human and civet S proteins	● Abs that neutralized most human S glycoproteins enhanced entry mediated by the civet virus S glycoproteins. The mechanism of enhancement involved the interaction of Abs with conformational epitopes in the hACE-2-binding domain<br>Entry of SARS-CoV can be enhanced by Abs.

De Groot, 2003[107]	2003	United States	Review	Varies	SARS-CoV and HIV	● Antibody seroconversion occurs around day 10.<br>Observed antibody-mediated exacerbation in feline and bovine coronaviruses.<br>SARS-CoV, like HIV, is an RNA virus that has an error-prone replication mechanism, which may explain mutations in the SARS-CoV genome in the S protein along with response to immune pressure.
Subbarao et al., 2004[108]	2003	United States	Mouse model study	Female BALBc mice 4-6 weeks old	SARS-CoV, neutralization assay	● Challenge with SARS-CoV revealed primary infection provided high levels of resistance to replication of the challenge virus or immune serum.<br>Neutralizing Abs were developed 28 days later.<br>Rapid time course for SARS-CoV replication in mice.
Weiss & Scott, 1981[109]	1980	United States	Serum challenge Study	12 week old specific-pathogen-free kittens	Feline infectious peritonitis virus (FIPV),IF,	● Non-immune kittens passively immunized with high titer serum developed enhanced disease.<br>Kittens with FIPV antibodies developed clinical signs earlier and died more rapidly compared to kittens non-sensitized that did not show clinical signs or die of FIPV revealing evidence of ADE.
Yang et al., 2004[110]	2004	United States	Vaccine Study	Female BALBc mice 6-8 weeks old<br>Protein expression was confirmed with recovered patients	SARS-CoV, ELISA	● DNA vaccine encoding the S protein of SARS-CoV induces T cell and neutralizing antibodies as well as protective immunity.<br>Mice vaccinated with an expression vector encoding S elicited neutralizing antibodies.

Rockx et al. 2008[68]	2008	United States	Mouse challenge study	Passive immunization was conducted on 10-12 week old BALBc mice<br>Human monoclonal antibody generation were described in:Traggiai et al. (2004)	SARS-CoV, neutralization, ELISA,	● The majority of human mAbs recognize a set of overlapping epitopes, since reactivity was lost by denaturation of the antigen (as seen in a cited hepatitis B study).<br>Escape mutant analysis to identify key residues in neutralizing activity of two cross-neutralizing mAb.
Dawson et al., 2019[111]	2019	United States	Review	n=407 articles reviewed and n=208 included	MERS-CoV, various diagnostic assays	● The main route of entry for MERS is di-peptidyl peptidase 4 (CD26) and like SARS-CoV uses a spike protein as the RBD.

Supplementary Table 7. Summary of studies on population-level seroprevalence of CoV.

**Author, Year Published**	**Virus**	**Assay**	**Figures Digitized**	**Notes**
Dijkman et al, 2008[35]	HCoVs NL63 and 229E	ELISA	n/a	Finds that majority of HCoV-NL63 seroconversion occurs before 3.5 years of age
Severance et al, 2009[86]	HCoVs 229E, HKU1, NL63, OC43	ELISA	Table 1	Cross-sectional survey of children (10) and adults (96)
Dijkman et al, 2012[42]	HCoVs 229E, HKU1, NL63, OC44	ELISA	Table 1	Longitudinal follow-up of 25 healthy infants from birth to 24 months
Müller et al, 2015[112]	MERS	ELISA IgG, IFA, neutralization	Supplement	Cross-sectional serosurvey for MERS in Saudi Arabia from general population
Chan et al, 2004[113]	SARS	IgG (assay unclear)	Text	The authors attributed the seropositivity differences to some patients in the older age groups not available for convalescent blood sample (died/moved wards)
Falsey et al, 2002[114]	HCoV 229E and OC43	EIA for IgG	n/a	Respiratory infection hospitalization surveillance
Sarateanu et al, 1980[115]	HCoV OC43	HI	Table 1	Serosurveillance over 2 years in Hamburg, Germany
Hovi et al, 1979[116]	HCoV OC43	CF, HI, RIA	n/a	Serosurvey of CoV across age groups and assays
Walsh et al, 2013[117]	HCoV 229E and OC43	EIA	Table 2	Seroincidence of three cohorts varying in age and health status

Gao et al, 2016[59]	Six HCoVs (incl SARS)	ELISA/WB	n/a	Examines cross-reactivity between HCoVs
Ukkonen et al, 1984[118]	HCoV OC43	CF	n/a	Complement fixation antibody responses across 16 respiratory pathogens of 58,000+ patients and 8 years
Liang et al, 2013[65]	HCoV OC43	WB	Table 1	HCoV exploration of N protein and immunoreactivity
Cavallaro et al, 1971[119]	HCoV 229E	CF and neutralization	Figures 2 & 3	Population level surveillance of 229E already in place during a 229E outbreak in Tecumseh, Michigan
Monto et al, 1974[83]	HCoV OC43	HAI and CF	Tables 1 & 3	Population level surveillance of OC43 already in place during a 229E outbreak in Tecumseh, Michigan
Degnah et al, 2020[120]	MERS	ELISA, ppNT, MNT	Table 3	Seroprevalence of MERS in Saudi Arabia indicating possible asymptomatic infections
Zhou et al, 2013[121]	HCoV 229E, OC43, NL63, HKU1	IFA for IgG and IgM	Figures 3 & 4	Serosurvey providing further evidence of early onset of first infection
Chan et al, 2009[122]	HCoV HKU1	anti-S ELISA+WB	Figure 6	Assay development and testing across CoV serum samples
Shao et al, 2007[123]	HCoV 229E and NL63	ELISA	Table 1	Seroprevalence study among individuals <20 years old
Cereda et al, 1986[124]	HCoV OC43 and 229E	indirect immuno-peroxidase staining	n/a	Seroprevalence among individuals hospitalized for any cause
Schmidt et al, 1986[125]	HCoV OC43 and 229E	ELISA IgG	Table 2	Seroprevalence lower among adults in 10 families in Seattle

Supplementary Table 8. Estimated annual force of infection from digitized age-seroprevalence data for endemic HCoVs

Strain	Author, Year Published	Annual force of infection (95% CI)
HCoV-229E	Zhou et al, 2013[121]	0.06 (0.03,0.10)
	Shao et al, 2007[123]	0.16 (0.09,0.26)
	Severance et al, 2009[86]	0.06 (0.04,0.09)
	Cavallaro et al, 1971[119]	0.01 (0.008,0.02)
HCoV-OC43	Zhou et al, 2013[121]	0.05 (0.03,0.09)
	Severance et al, 2009[86]	0.06 (0.04,0.09)
	Sarateanu et al, 1980[115]	0.03 (0.01,0.05)
HCoV-NL63	Zhou et al, 2013[121]	0.04 (0.02,0.08)
	Shao et al, 2007[123]	0.12 (0.09,0.16)
	Severance et al, 2009[86]	0.06 (0.04,0.09)
HCoV-HKU1	Zhou et al, 2013[121]	0.05 (0.02,0.08)
	Severance et al, 2009[86]	0.02 (0.01,0.03)
	Chan et al, 2009[122]	0.004 (0.003,0.006)

Supplementary Figures

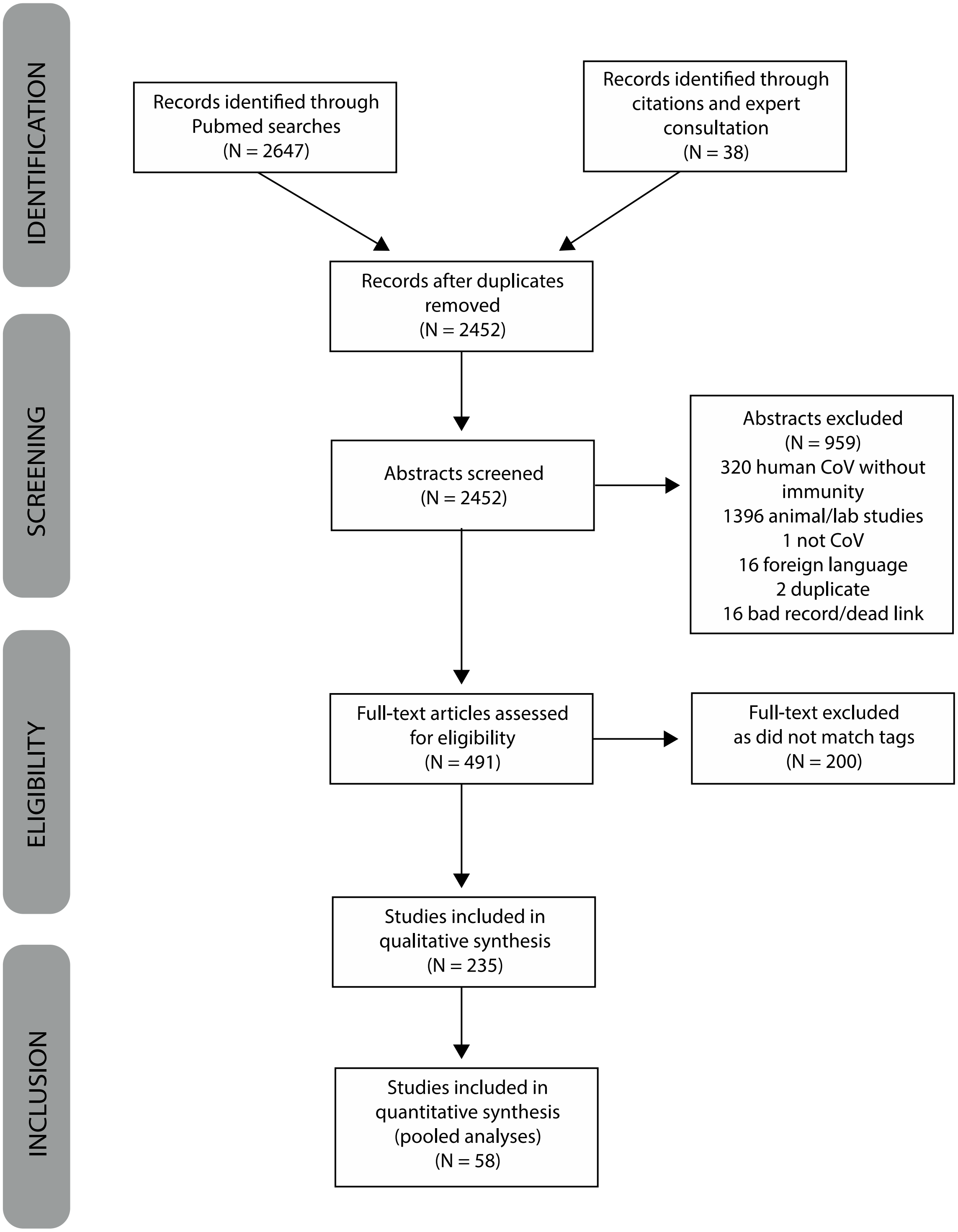

Supplementary Figure 1. PRISMA diagram of systematic review.

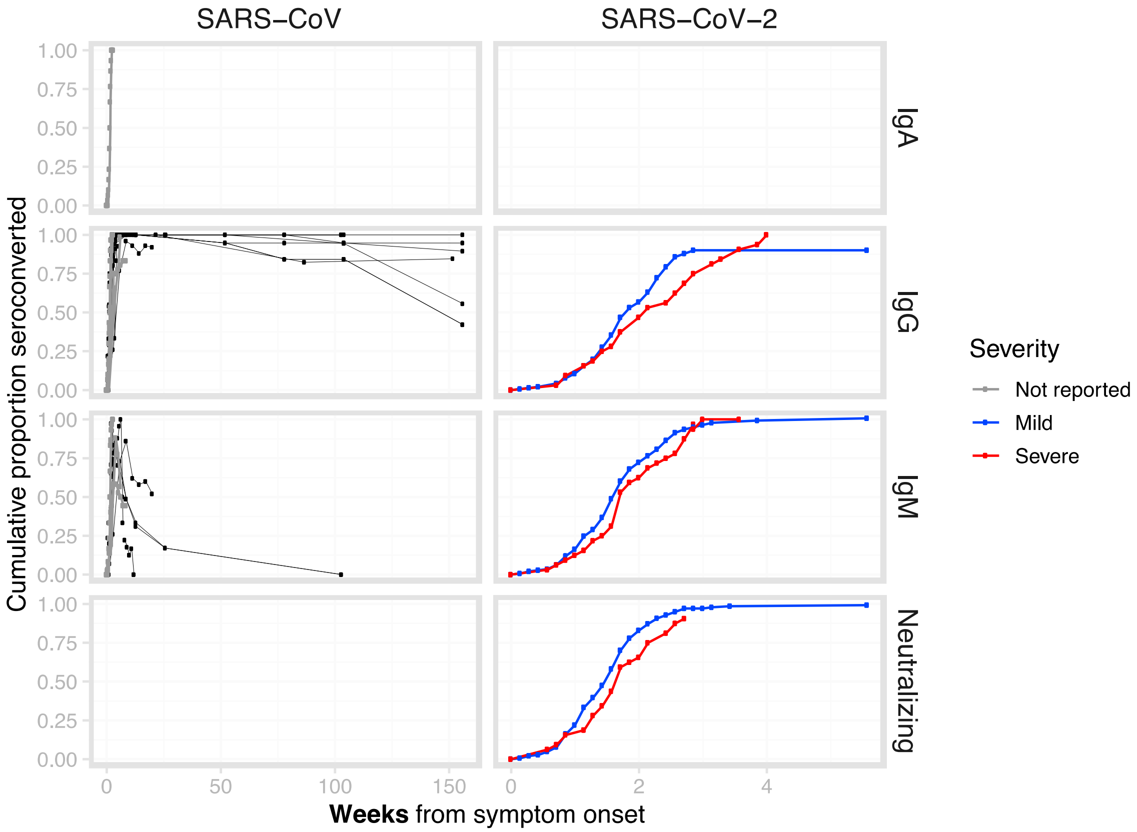

Supplementary Figure 2.

Cumulative proportion of patients that seroconverted from four studies digitized separately by severity. Studies on SARS-CoV did not provide information on severity of symptoms. Black points and lines show non-cumulative proportions of patients that seroconverted at respective times (upper bound if reported as time intervals) from thirteen other studies.

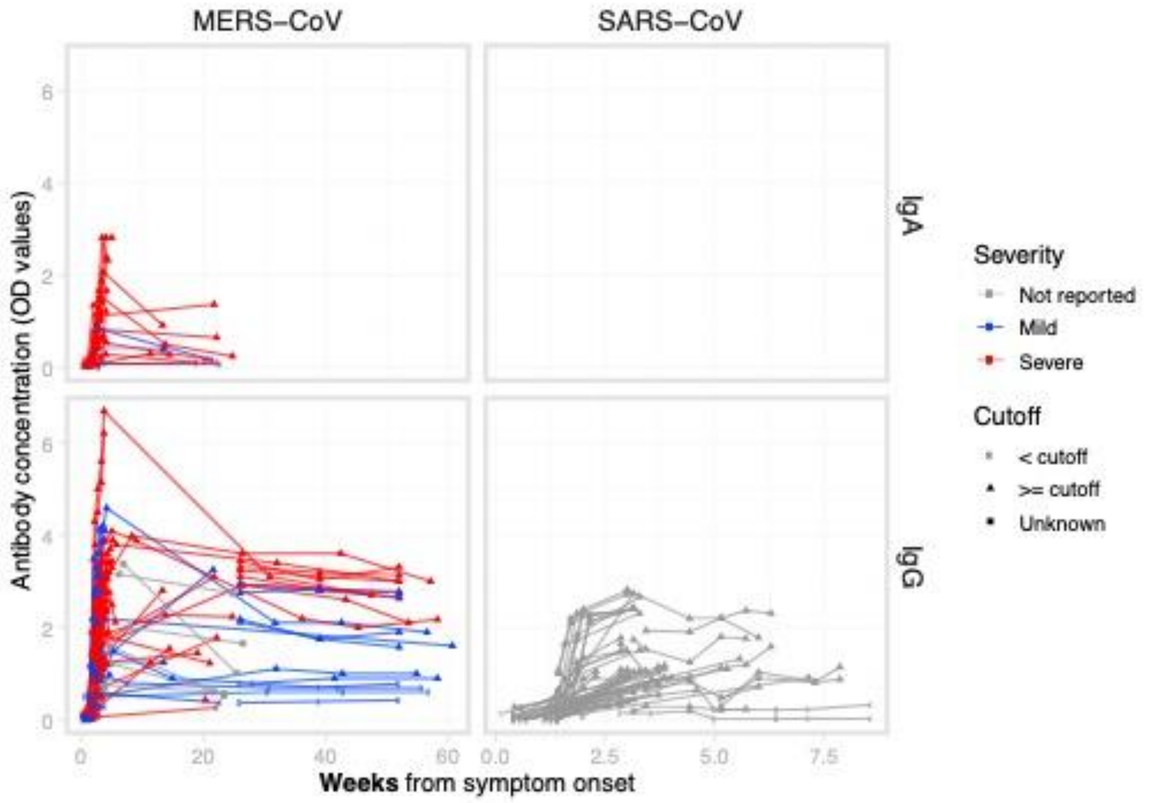

Supplementary Figure 3.
Antibody time series reported in studies in units of optical density (OD). Some studies may report time series of different antibodies for the same patient. The plotting symbols indicate whether a measurement was above the cutoff for the assay being used, if reported in the study. Note that while these are plotted on the same axis, values may not necessarily be compared across studies as each may employ different scales.

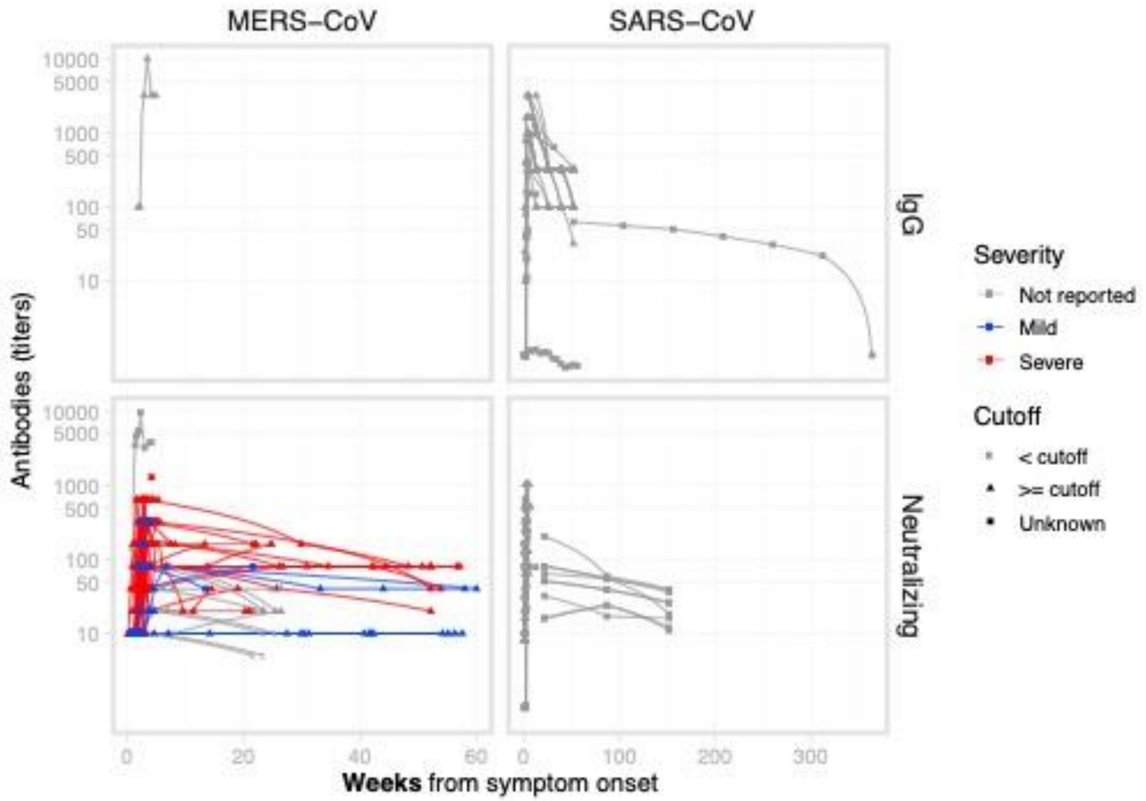

Supplementary Figure 4.
Antibody time series reported in studies in units of titers. Some studies may report time series of different antibodies or using different assays for the same patient. The plotting symbols indicate whether a measurement was above the cutoff for the assay being used, if reported in the study. Some studies reported titers that were lower than or greater than some threshold value; those are here plotted at those values (e.g., for ≥ 320, the value is assumed to be 320). Note that while these are plotted on the same axis, values may not necessarily be compared across studies as each may use different scales.

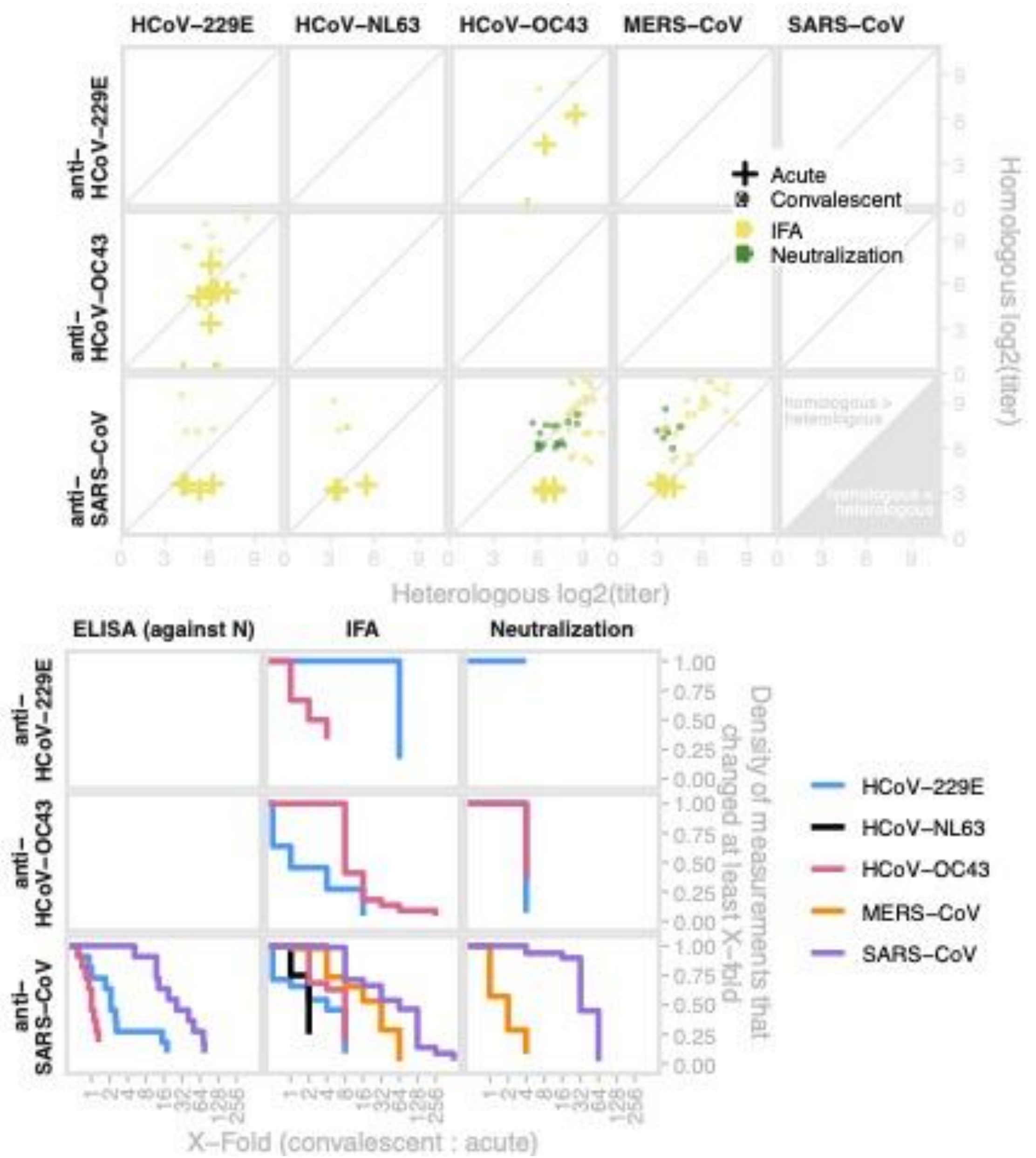

Supplementary Figure 5.
Reactivity of antisera against homotypic and heterotypic HCoVs post infection. (Top) Reactivity of antiserum (x-axis) taken from individuals with confirmed infections of each human coronavirus against a panel of human coronaviruses (columns) shown in relation to their phylogeny as measured by IFA (yellow) and neutralization (green); convalescent (circles) and acute sera (crosses). The y-axis provides titers to the homologous strain of the sera (rows) for comparison; points have been jittered to reveal observations stacked at the same position. (Bottom) Density of reported convalescent-to-acute titer fold-rise upon infection (y-axis) which were at least of a particular value (x-axis). Data extracted from seven studies[18,39,40,43–46].

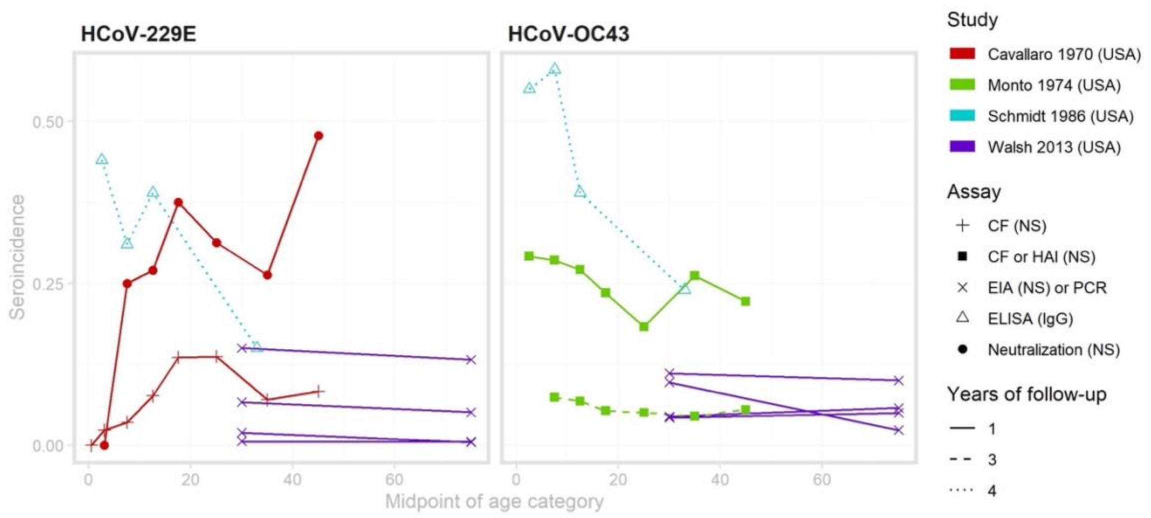

Supplementary Figure 6.

Age-incidence curves for studies with appropriate, digitizable data on endemic HCoV. The color denotes the study, the point type denotes the assay and antibody measured, and the line type represents the number of years between successive serosamples [18,39,40,43–46].

Supplementary References

1. Riski, H. & Hovi, T. Coronavirus infections of man associated with diseases other than the common cold. *J. Med. Virol.* **6**, 259–265 (1980).
2. Kraaijeveld, C. A., Reed, S. E. & Macnaughton, M. R. Enzyme-linked immunosorbent assay for detection of antibody in volunteers experimentally infected with human coronavirus strain 229 E. *J. Clin. Microbiol.* **12**, 493–497 (1980).
3. Callow, K. A., Parry, H. F., Sergeant, M. & Tyrrell, D. A. The time course of the immune response to experimental coronavirus infection of man. *Epidemiol. Infect.* **105**, 435–446 (1990).
4. Azhar, E. I. *et al.* Evidence for camel-to-human transmission of MERS coronavirus. *N. Engl. J. Med.* **370**, 2499–2505 (2014).
5. Okba, N. M. A. *et al.* Sensitive and Specific Detection of Low-Level Antibody Responses in Mild Middle East Respiratory Syndrome Coronavirus Infections. *Emerg. Infect. Dis.* **25**, 1868–1877 (2019).
6. Alshukairi, A. N. *et al.* Antibody Response and Disease Severity in Healthcare Worker MERS Survivors. *Emerg. Infect. Dis.* **22**, (2016).
7. Spanakis, N. *et al.* Virological and serological analysis of a recent Middle East respiratory syndrome coronavirus infection case on a triple combination antiviral regimen. *Int. J. Antimicrob. Agents* **44**, 528–532 (2014).
8. Park, W. B. *et al.* Kinetics of Serologic Responses to MERS Coronavirus Infection in Humans, South Korea. *Emerg. Infect. Dis.* **21**, 2186–2189 (2015).
9. Wang, W. L. *et al.* Serological Study of An Imported Case of Middle East Respiratory Syndrome and His Close Contacts in China, 2015. *Biomed. Environ. Sci.* **29**, 219–223 (2016).
10. Ko, J.-H. *et al.* Serologic responses of 42 MERS-coronavirus-infected patients according to the disease severity. *Diagn. Microbiol. Infect. Dis.* **89**, 106–111 (2017).
11. Choe, P. G. *et al.* MERS-CoV Antibody Responses 1 Year after Symptom Onset, South Korea, 2015. *Emerg. Infect. Dis.* **23**, 1079–1084 (2017).
12. Al-Abdely, H. M. *et al.* Middle East Respiratory Syndrome Coronavirus Infection Dynamics and Antibody Responses among Clinically Diverse Patients, Saudi Arabia. *Emerg. Infect. Dis.* **25**, 753–766 (2019).
13. Van Kerkhove, M. D. *et al.* Transmissibility of MERS-CoV Infection in Closed Setting, Riyadh, Saudi Arabia, 2015. *Emerg. Infect. Dis.* **25**, 1802–1809 (2019).
14. Hsueh, P.-R. *et al.* Microbiologic characteristics, serologic responses, and clinical manifestations in severe acute respiratory syndrome, Taiwan. *Emerg. Infect. Dis.* **9**, 1163–1167 (2003).
15. Wu, H.-S. *et al.* Serologic and molecular biologic methods for SARS-associated coronavirus infection, Taiwan. *Emerg. Infect. Dis.* **10**, 304–310 (2004).
16. Chen, X. *et al.* Serology of severe acute respiratory syndrome: implications for surveillance and outcome. *J. Infect. Dis.* **189**, 1158–1163 (2004).
17. Tsao, K.-C. *et al.* False positive antibody results against human T-cell lymphotropic virus in patients with severe acute respiratory syndrome. *J. Med. Virol.* **77**, 331–336 (2005).
18. Chan, K. H. *et al.* Serological responses in patients with severe acute respiratory syndrome coronavirus infection and cross-reactivity with human coronaviruses 229E, OC43, and NL63. *Clin. Diagn. Lab. Immunol.* **12**, 1317–1321 (2005).
19. He, W.-P., Shu, C.-L., Li, B.-A., Zhao, J. & Cheng, Y. Human LINE1 endonuclease domain as a putative target of SARS-associated autoantibodies involved in the pathogenesis of severe acute respiratory syndrome. *Chin. Med. J.* **121**, 608–614 (2008).
20. Liao, J.-W. *et al.* A retrospective serological study of severe acute respiratory syndrome cases in

Guangdong province, China. *Chin. Med. J.* **120**, 718–720 (2007).
21. Cao, W.-C., Liu, W., Zhang, P.-H., Zhang, F. & Richardus, J. H. Disappearance of antibodies to SARS-associated coronavirus after recovery. *N. Engl. J. Med.* **357**, 1162–1163 (2007).
22. Hsueh, P.-R., Huang, L.-M., Chen, P.-J., Kao, C.-L. & Yang, P.-C. Chronological evolution of IgM, IgA, IgG and neutralisation antibodies after infection with SARS-associated coronavirus. *Clin. Microbiol. Infect.* **10**, 1062–1066 (2004).
23. Yang, Z. *et al.* Determining SARS sub-clinical infection: a longitudinal seroepidemiological study in recovered SARS patients and controls after an outbreak in a general hospital. *Scand. J. Infect. Dis.* **41**, 507–510 (2009).
24. Tang, F. *et al.* Lack of peripheral memory B cell responses in recovered patients with severe acute respiratory syndrome: a six-year follow-up study. *J. Immunol.* **186**, 7264–7268 (2011).
25. Zhang, W. *et al.* Molecular and serological investigation of 2019-nCoV infected patients: implication of multiple shedding routes. *Emerg. Microbes Infect.* **9**, 386–389 (2020).
26. Zhao, J. *et al.* Antibody Responses to SARS-CoV-2 in Patients of Novel Coronavirus Disease 2019. *Preprint with The Lancet* (2020).
27. Tan, W. *et al.* Viral Kinetics and Antibody Responses in Patients with COVID-19. *medRxiv* (2020).
28. Mo, H. *et al.* Longitudinal profile of antibodies against SARS-coronavirus in SARS patients and their clinical significance. *Respirology* **11**, 49–53 (2006).
29. Zhuang J.-H., Huang X.-Z., Zhou Q., Lin L.-Y. & Lin L. [Dynamic observation IgG and IgM antibodies in patients with severe acute respiratory syndrome]. *Zhongguo Wei Zhong Bing Ji Jiu Yi Xue* **15**, 579–581 (2003).
30. Liu Y.-N., Fan B.-X., Fang X.-Q., Yu B.-X. & Chen L.-A. [The quantitative detection of anti-coronavirus antibody titer in medical personnel closely contacted with severe acute respiratory syndrome patients]. *Zhonghua Jie He He Hu Xi Za Zhi* **26**, 583–585 (2003).
31. Abbasi, S. *et al.* Can low-dose of ketamine reduce the need for morphine in renal colic? A double-blind randomized clinical trial. *Am. J. Emerg. Med.* **36**, 376–379 (2018).
32. Zhao, J. *et al.* Antibody responses to SARS-CoV-2 in patients of novel coronavirus disease 2019. *Clin. Infect. Dis.* (2020) doi:10.1093/cid/ciaa344.
33. Callow, K. A. Effect of specific humoral immunity and some non-specific factors on resistance of volunteers to respiratory coronavirus infection. *J. Hyg.* **95**, 173–189 (1985).
34. Hamre, D. & Beem, M. Virologic studies of acute respiratory disease in young adults. V. Coronavirus 229E infections during six years of surveillance. *Am. J. Epidemiol.* **96**, 94–106 (1972).
35. Dijkman, R. *et al.* Human coronavirus NL63 and 229E seroconversion in children. *J. Clin. Microbiol.* **46**, 2368–2373 (2008).
36. Reed, S. E. The behaviour of recent isolates of human respiratory coronavirus in vitro and in volunteers: evidence of heterogeneity among 229E-related strains. *J. Med. Virol.* **13**, 179–192 (1984).
37. Cohen, S., Tyrrell, D. A. & Smith, A. P. Psychological stress and susceptibility to the common cold. *N. Engl. J. Med.* **325**, 606–612 (1991).
38. Barrow, G. I. *et al.* The effect of intranasal nedocromil sodium on viral upper respiratory tract infections in human volunteers. *Clin. Exp. Allergy* **20**, 45–51 (1990).
39. Bradburne, A. F. Antigenic relationships amongst coronaviruses. *Arch. Gesamte Virusforsch.* **31**, 352–364 (1970).
40. Kaye, H. S., Ong, S. B. & Dowdle, W. R. Detection of coronavirus 229E antibody by indirect hemagglutination. *Appl. Microbiol.* **24**, 703–707 (1972).
41. Macnaughton, M. R., Madge, M. H. & Reed, S. E. Two antigenic groups of human coronaviruses detected by using enzyme-linked immunosorbent assay. *Infect. Immun.* **33**, 734–737 (1981).

42. Dijkman, R. *et al.* The dominance of human coronavirus OC43 and NL63 infections in infants. *J. Clin. Virol.* **53**, 135–139 (2012).
43. Lehmann, C. *et al.* A line immunoassay utilizing recombinant nucleocapsid proteins for detection of antibodies to human coronaviruses. *Diagn. Microbiol. Infect. Dis.* **61**, 40–48 (2008).
44. Haynes, L. M. *et al.* Recombinant protein-based assays for detection of antibodies to severe acute respiratory syndrome coronavirus spike and nucleocapsid proteins. *Clin. Vaccine Immunol.* **14**, 331–333 (2007).
45. Che, X.-Y. *et al.* Antigenic cross-reactivity between severe acute respiratory syndrome-associated coronavirus and human coronaviruses 229E and OC43. *J. Infect. Dis.* **191**, 2033–2037 (2005).
46. Chan, K.-H. *et al.* Cross-reactive antibodies in convalescent SARS patients' sera against the emerging novel human coronavirus EMC (2012) by both immunofluorescent and neutralizing antibody tests. *J. Infect.* **67**, 130–140 (2013).
47. Liang, Y. *et al.* Comprehensive antibody epitope mapping of the nucleocapsid protein of severe acute respiratory syndrome (SARS) coronavirus: insight into the humoral immunity of SARS. *Clin. Chem.* **51**, 1382–1396 (2005).
48. Fung, T. S. & Liu, D. X. Human Coronavirus: Host-Pathogen Interaction. *Annu. Rev. Microbiol.* **73**, 529–557 (2019).
49. Zhong, X. *et al.* B-cell responses in patients who have recovered from severe acute respiratory syndrome target a dominant site in the S2 domain of the surface spike glycoprotein. *J. Virol.* **79**, 3401–3408 (2005).
50. Cui, J., Li, F. & Shi, Z.-L. Origin and evolution of pathogenic coronaviruses. *Nat. Rev. Microbiol.* **17**, 181–192 (2019).
51. Ren, Y. *et al.* A strategy for searching antigenic regions in the SARS-CoV spike protein. *Genomics Proteomics Bioinformatics* **1**, 207–215 (2003).
52. Bisht, H. *et al.* Severe acute respiratory syndrome coronavirus spike protein expressed by attenuated vaccinia virus protectively immunizes mice. *Proc. Natl. Acad. Sci. U. S. A.* **101**, 6641–6646 (2004).
53. Wang, Y. *et al.* Profiles of IgG antibodies to nucleocapsid and spike proteins of the SARS-associated coronavirus in SARS patients. *DNA Cell Biol.* **24**, 521–527 (2005).
54. Meyer, B. *et al.* Antibodies against MERS coronavirus in dromedary camels, United Arab Emirates, 2003 and 2013. *Emerg. Infect. Dis.* **20**, 552–559 (2014).
55. Patrick, D. M. *et al.* An Outbreak of Human Coronavirus OC43 Infection and Serological Cross-reactivity with SARS Coronavirus. *Can. J. Infect. Dis. Med. Microbiol.* **17**, 330–336 (2006).
56. Du, L., Ma, C. & Jiang, S. Antibodies induced by receptor-binding domain in spike protein of SARS-CoV do not cross-neutralize the novel human coronavirus hCoV-EMC. *The Journal of infection* vol. 67 348–350 (2013).
57. Aburizaiza, A. S. *et al.* Investigation of anti-middle East respiratory syndrome antibodies in blood donors and slaughterhouse workers in Jeddah and Makkah, Saudi Arabia, fall 2012. *J. Infect. Dis.* **209**, 243–246 (2014).
58. Al Kahlout, R. A. *et al.* Comparative Serological Study for the Prevalence of Anti-MERS Coronavirus Antibodies in High- and Low-Risk Groups in Qatar. *J Immunol Res* **2019**, 1386740 (2019).
59. Gao, X. *et al.* Antibody against nucleocapsid protein predicts susceptibility to human coronavirus infection. *J. Infect.* **71**, 599–602 (2015).
60. Trivedi, S. U. *et al.* Development and Evaluation of a Multiplexed Immunoassay for Simultaneous Detection of Serum IgG Antibodies to Six Human Coronaviruses. *Sci. Rep.* **9**, 1390 (2019).
61. Agnihothram, S. *et al.* Evaluation of serologic and antigenic relationships between middle eastern respiratory syndrome coronavirus and other coronaviruses to develop vaccine platforms for the rapid

response to emerging coronaviruses. *J. Infect. Dis.* **209**, 995–1006 (2014).
62. Vlasova, A. N. *et al.* Two-way antigenic cross-reactivity between severe acute respiratory syndrome coronavirus (SARS-CoV) and group 1 animal CoVs is mediated through an antigenic site in the N-terminal region of the SARS-CoV nucleoprotein. *J. Virol.* **81**, 13365–13377 (2007).
63. Yu, F. *et al.* Evaluation of inapparent nosocomial severe acute respiratory syndrome coronavirus infection in Vietnam by use of highly specific recombinant truncated nucleocapsid protein-based enzyme-linked immunosorbent assay. *Clin. Diagn. Lab. Immunol.* **12**, 848–854 (2005).
64. Mu, F. *et al.* The expression and antigenicity of a truncated spike-nucleocapsid fusion protein of severe acute respiratory syndrome-associated coronavirus. *BMC Microbiol.* **8**, 207 (2008).
65. Liang, F.-Y. *et al.* Immunoreactivity characterisation of the three structural regions of the human coronavirus OC43 nucleocapsid protein by Western blot: implications for the diagnosis of coronavirus infection. *J. Virol. Methods* **187**, 413–420 (2013).
66. Carattoli, A. *et al.* Recombinant protein-based ELISA and immuno-cytochemical assay for the diagnosis of SARS. *J. Med. Virol.* **76**, 137–142 (2005).
67. He, Y., Zhou, Y., Siddiqui, P., Niu, J. & Jiang, S. Identification of immunodominant epitopes on the membrane protein of the severe acute respiratory syndrome-associated coronavirus. *J. Clin. Microbiol.* **43**, 3718–3726 (2005).
68. Rockx, B. *et al.* Structural basis for potent cross-neutralizing human monoclonal antibody protection against lethal human and zoonotic severe acute respiratory syndrome coronavirus challenge. *J. Virol.* **82**, 3220–3235 (2008).
69. Zhu, Z. *et al.* Potent cross-reactive neutralization of SARS coronavirus isolates by human monoclonal antibodies. *Proc. Natl. Acad. Sci. U. S. A.* **104**, 12123–12128 (2007).
70. Liu, L. *et al.* Natural mutations in the receptor binding domain of spike glycoprotein determine the reactivity of cross-neutralization between palm civet coronavirus and severe acute respiratory syndrome coronavirus. *J. Virol.* **81**, 4694–4700 (2007).
71. He, Y. *et al.* Cross-neutralization of human and palm civet severe acute respiratory syndrome coronaviruses by antibodies targeting the receptor-binding domain of spike protein. *J. Immunol.* **176**, 6085–6092 (2006).
72. Elshabrawy, H. A., Coughlin, M. M., Baker, S. C. & Prabhakar, B. S. Human monoclonal antibodies against highly conserved HR1 and HR2 domains of the SARS-CoV spike protein are more broadly neutralizing. *PLoS One* **7**, e50366 (2012).
73. Tian, X. *et al.* Potent binding of 2019 novel coronavirus spike protein by a SARS coronavirus-specific human monoclonal antibody. *Emerg. Microbes Infect.* **9**, 382–385 (2020).
74. Tai, W. *et al.* Recombinant Receptor-Binding Domains of Multiple Middle East Respiratory Syndrome Coronaviruses (MERS-CoVs) Induce Cross-Neutralizing Antibodies against Divergent Human and Camel MERS-CoVs and Antibody Escape Mutants. *J. Virol.* **91**, (2017).
75. Yu, Y. *et al.* Children's vaccines do not induce cross reactivity against SARS-CoV. *J. Clin. Pathol.* **60**, 208–211 (2007).
76. Resta, S., Luby, J. P., Rosenfeld, C. R. & Siegel, J. D. Isolation and propagation of a human enteric coronavirus. *Science* **229**, 978–981 (1985).
77. Gerna, G., Passarani, N., Cereda, P. M. & Battaglia, M. Antigenic relatedness of human enteric coronavirus strains to human coronavirus OC43: a preliminary report. *J. Infect. Dis.* **150**, 618–619 (1984).
78. Han, M. G., Cheon, D.-S., Zhang, X. & Saif, L. J. Cross-protection against a human enteric coronavirus and a virulent bovine enteric coronavirus in gnotobiotic calves. *J. Virol.* **80**, 12350–12356 (2006).
79. Gerdes, J. C., Klein, I., DeVald, B. L. & Burks, J. S. Coronavirus isolates SK and SD from multiple sclerosis

patients are serologically related to murine coronaviruses A59 and JHM and human coronavirus OC43, but not to human coronavirus 229E. *J. Virol.* **38**, 231–238 (1981).

80. Kaye, H. S., Yarbrough, W. B., Reed, C. J. & Harrison, A. K. Antigenic relationship between human coronavirus strain OC 43 and hemagglutinating encephalomyelitis virus strain 67N of swine: antibody responses in human and animal sera. *J. Infect. Dis.* **135**, 201–209 (1977).
81. Schmidt, O. W. & Kenny, G. E. Immunogenicity and antigenicity of human coronaviruses 229E and OC43. *Infect. Immun.* **32**, 1000–1006 (1981).
82. McIntosh, K. *et al.* Seroepidemiologic studies of coronavirus infection in adults and children. *Am. J. Epidemiol.* **91**, 585–592 (1970).
83. Monto, A. S. & Lim, S. K. The Tecumseh study of respiratory illness. VI. Frequency of and relationship between outbreaks of coronavirus infection. *J. Infect. Dis.* **129**, 271–276 (1974).
84. Tuan, P. A. *et al.* SARS transmission in Vietnam outside of the health-care setting. *Epidemiol. Infect.* **135**, 392–401 (2007).
85. Richardson, S. E., Tellier, R. & Mahony, J. The laboratory diagnosis of severe acute respiratory syndrome: emerging laboratory tests for an emerging pathogen. *Clin. Biochem. Rev.* **25**, 133–141 (2004).
86. Severance, E. G. *et al.* Development of a nucleocapsid-based human coronavirus immunoassay and estimates of individuals exposed to coronavirus in a U.S. metropolitan population. *Clin. Vaccine Immunol.* **15**, 1805–1810 (2008).
87. Kossyvakis, A. *et al.* Laboratory investigation and phylogenetic analysis of an imported Middle East respiratory syndrome coronavirus case in Greece. *PLoS One* **10**, e0125809 (2015).
88. Peiris, J. S. M. *et al.* Clinical progression and viral load in a community outbreak of coronavirus-associated SARS pneumonia: a prospective study. *Lancet* **361**, 1767–1772 (2003).
89. Ho, M.-S. *et al.* Neutralizing antibody response and SARS severity. *Emerg. Infect. Dis.* **11**, 1730–1737 (2005).
90. Cameron, M. J., Bermejo-Martin, J. F., Danesh, A., Muller, M. P. & Kelvin, D. J. Human immunopathogenesis of severe acute respiratory syndrome (SARS). *Virus Res.* **133**, 13–19 (2008).
91. Talbot, H. K. *et al.* The pediatric burden of human coronaviruses evaluated for twenty years. *Pediatr. Infect. Dis. J.* **28**, 682–687 (2009).
92. Jaume, M. *et al.* Anti-severe acute respiratory syndrome coronavirus spike antibodies trigger infection of human immune cells via a pH- and cysteine protease-independent FcγR pathway. *J. Virol.* **85**, 10582–10597 (2011).
93. Yip, M. S. *et al.* Antibody-dependent infection of human macrophages by severe acute respiratory syndrome coronavirus. *Virol. J.* **11**, 82 (2014).
94. Fu, Y., Cheng, Y. & Wu, Y. Understanding SARS-CoV-2-Mediated Inflammatory Responses: From Mechanisms to Potential Therapeutic Tools. *Virol. Sin.* (2020) doi:10.1007/s12250-020-00207-4.
95. Wang, S.-F. *et al.* Antibody-dependent SARS coronavirus infection is mediated by antibodies against spike proteins. *Biochem. Biophys. Res. Commun.* **451**, 208–214 (2014).
96. Yip, M. S. *et al.* Antibody-dependent enhancement of SARS coronavirus infection and its role in the pathogenesis of SARS. *Hong Kong Med. J.* **22**, 25–31 (2016).
97. Perlman, S. & Dandekar, A. A. Immunopathogenesis of coronavirus infections: implications for SARS. *Nat. Rev. Immunol.* **5**, 917–927 (2005).
98. Lin, Y. S. *et al.* Antibody to severe acute respiratory syndrome (SARS)-associated coronavirus spike protein domain 2 cross-reacts with lung epithelial cells and causes cytotoxicity. *Clin. Exp. Immunol.* **141**, 500–508 (2005).
99. Sun, W. *et al.* Osteonecrosis in patients after severe acute respiratory syndrome (SARS): possible role

of anticardiolipin antibodies. *J. Clin. Rheumatol.* **16**, 61–63 (2010).

100. Fang, Y.-T. *et al.* Annexin A2 on lung epithelial cell surface is recognized by severe acute respiratory syndrome-associated coronavirus spike domain 2 antibodies. *Mol. Immunol.* **47**, 1000–1009 (2010).
101. Cheng, M. *et al.* Cross-reactivity of antibody against SARS-coronavirus nucleocapsid protein with IL-11. *Biochem. Biophys. Res. Commun.* **338**, 1654–1660 (2005).
102. Yasmon, A., Ibrahim, F., Bela, B. & Sjahrurachman, A. Potential cross-reactivity of severe acute respiratory syndrome-associated coronavirus (SARS-CoV) nucleocapsid (N)-based IgG ELISA assay for plasma samples from HIV-1 positive intravenous drug users (IDUs). *Acta Med. Indones.* **44**, 193–198 (2012).
103. Ksiazek, T. G. *et al.* A novel coronavirus associated with severe acute respiratory syndrome. *N. Engl. J. Med.* **348**, 1953–1966 (2003).
104. Gorse, G. J., Donovan, M. M. & Patel, G. B. Antibodies to coronaviruses are higher in older compared with younger adults and binding antibodies are more sensitive than neutralizing antibodies in identifying coronavirus-associated illnesses. *J. Med. Virol.* **92**, 512–517 (2020).
105. Bermingham, A. *et al.* Laboratory diagnosis of SARS. *Philos. Trans. R. Soc. Lond. B Biol. Sci.* **359**, 1083–1089 (2004).
106. Yang, Z.-Y. *et al.* Evasion of antibody neutralization in emerging severe acute respiratory syndrome coronaviruses. *Proc. Natl. Acad. Sci. U. S. A.* **102**, 797–801 (2005).
107. De Groot, A. S. How the SARS vaccine effort can learn from HIV-speeding towards the future, learning from the past. *Vaccine* **21**, 4095–4104 (2003).
108. Subbarao, K. *et al.* Prior infection and passive transfer of neutralizing antibody prevent replication of severe acute respiratory syndrome coronavirus in the respiratory tract of mice. *J. Virol.* **78**, 3572–3577 (2004).
109. Weiss, R. C. & Scott, F. W. Antibody-mediated enhancement of disease in feline infectious peritonitis: comparisons with dengue hemorrhagic fever. *Comp. Immunol. Microbiol. Infect. Dis.* **4**, 175–189 (1981).
110. Yang, Z.-Y. *et al.* A DNA vaccine induces SARS coronavirus neutralization and protective immunity in mice. *Nature* **428**, 561–564 (2004).
111. Dawson, P., Malik, M. R., Parvez, F. & Morse, S. S. What Have We Learned About Middle East Respiratory Syndrome Coronavirus Emergence in Humans? A Systematic Literature Review. *Vector Borne Zoonotic Dis.* **19**, 174–192 (2019).
112. Müller, M. A. *et al.* Presence of Middle East respiratory syndrome coronavirus antibodies in Saudi Arabia: a nationwide, cross-sectional, serological study. *Lancet Infect. Dis.* **15**, 559–564 (2015).
113. Chan, T. Y., Miu, K. Y., Tsui, C. K., Yee, K. S. & Chan, M. H. A comparative study of clinical features and outcomes in young and older adults with severe acute respiratory syndrome. *J. Am. Geriatr. Soc.* **52**, 1321–1325 (2004).
114. Falsey, A. R., Walsh, E. E. & Hayden, F. G. Rhinovirus and coronavirus infection-associated hospitalizations among older adults. *J. Infect. Dis.* **185**, 1338–1341 (2002).
115. Sarateanu, D. E. & Ehrengut, W. A two year serological surveillance of coronavirus infections in Hamburg. *Infection* **8**, 70–72 (1980).
116. Hovi, T., Kainulainen, H., Ziola, B. & Salmi, A. OC43 strain-related coronavirus antibodies in different age groups. *J. Med. Virol.* **3**, 313–320 (1979).
117. Walsh, E. E., Shin, J. H. & Falsey, A. R. Clinical impact of human coronaviruses 229E and OC43 infection in diverse adult populations. *J. Infect. Dis.* **208**, 1634–1642 (2013).
118. Ukkonen, P., Hovi, T., von Bonsdorff, C. H., Saikku, P. & Penttinen, K. Age-specific prevalence of complement-fixing antibodies to sixteen viral antigens: a computer analysis of 58,500 patients

covering a period of eight years. *J. Med. Virol.* **13**, 131–148 (1984).
119. Cavallaro, J. J. & Monto, A. S. Community-wide outbreak of infection with a 229E-like coronavirus in Tecumseh, Michigan. *J. Infect. Dis.* **122**, 272–279 (1970).
120. Degnah, A. A. *et al.* Seroprevalence of MERS-CoV in healthy adults in western Saudi Arabia, 2011-2016. *J. Infect. Public Health* **13**, 697–703 (2020).
121. Zhou, W., Wang, W., Wang, H., Lu, R. & Tan, W. First infection by all four non-severe acute respiratory syndrome human coronaviruses takes place during childhood. *BMC Infect. Dis.* **13**, 433 (2013).
122. Chan, C. M. *et al.* Examination of seroprevalence of coronavirus HKU1 infection with S protein-based ELISA and neutralization assay against viral spike pseudotyped virus. *J. Clin. Virol.* **45**, 54–60 (2009).
123. Shao, X., Guo, X., Esper, F., Weibel, C. & Kahn, J. S. Seroepidemiology of group I human coronaviruses in children. *J. Clin. Virol.* **40**, 207–213 (2007).
124. Cereda, P. M., Pagani, L. & Romero, E. Prevalence of antibody to human coronaviruses 229E, OC43 and neonatal calf diarrhea coronavirus (NCDCV) in patients of Northern Italy. *Eur. J. Epidemiol.* **2**, 112–117 (1986).
125. Schmidt, O. W., Allan, I. D., Cooney, M. K., Foy, H. M. & Fox, J. P. Rises in titers of antibody to human coronaviruses OC43 and 229E in Seattle families during 1975-1979. *Am. J. Epidemiol.* **123**, 862–868 (1986).
