# Supplementary material for: A systematic review of antibody mediated immunity to coronaviruses: kinetics, correlates of protection, and association with severity
Source: Nat Commun. 2020 Sep 17;11:4704. doi: 10.1038/s41467-020-18450-4 (PMC7499300; doi:10.1038/s41467-020-18450-4)
Supplement: Supplementary file 4 — Description of Additional Supplementary Files [file 41467_2020_18450_MOESM4_ESM.pdf]

Description of Additional Supplementary Files

File Name: Supplementary Data 1
Description: Data digitized on antibody kinetics and association of antibody responses with clinical severity.

File Name: Supplementary Data 2
Description: Data digitized on cross-reactivity and antigenic diversity.

File Name: Supplementary Data 3
Description: Data digitized on population seroprevalence.
